# Newly discovered Asgard archaea Hermodarchaeota potentially degrade alkanes and aromatics via alkyl/benzyl-succinate synthase and benzoyl-CoA pathway

**DOI:** 10.1038/s41396-020-00890-x

**Published:** 2021-01-15

**Authors:** Jia-Wei Zhang, Hong-Po Dong, Li-Jun Hou, Yang Liu, Ya-Fei Ou, Yan-Ling Zheng, Ping Han, Xia Liang, Guo-Yu Yin, Dian-Ming Wu, Min Liu, Meng Li

**Affiliations:** 1 State Key Laboratory of Estuarine and Coastal Research, East China Normal University, Shanghai, 200241 China; 2 School of Ocean and Meteorology, Guangdong Ocean University, Zhanjiang, 524088 China; 3 Shenzhen Key Laboratory of Marine Microbiome Engineering, Institute for Advanced Study, Shenzhen University, Shenzhen, 518060 China; 4 Key Laboratory of Geographic Information Science, Ministry of Education, East China Normal University, Shanghai, 200241 China

**Keywords:** Microbial ecology, Metagenomics

## Abstract

Asgard archaea are widely distributed in anaerobic environments. Previous studies revealed the potential capability of Asgard archaea to utilize various organic substrates including proteins, carbohydrates, fatty acids, amino acids and hydrocarbons, suggesting that Asgard archaea play an important role in sediment carbon cycling. Here, we describe a previously unrecognized archaeal phylum, Hermodarchaeota, affiliated with the Asgard superphylum. The genomes of these archaea were recovered from metagenomes generated from mangrove sediments, and were found to encode alkyl/benzyl-succinate synthases and their activating enzymes that are similar to those identified in alkane-degrading sulfate-reducing bacteria. Hermodarchaeota also encode enzymes potentially involved in alkyl-coenzyme A and benzoyl-coenzyme A oxidation, the Wood–Ljungdahl pathway and nitrate reduction. These results indicate that members of this phylum have the potential to strictly anaerobically degrade alkanes and aromatic compounds, coupling the reduction of nitrate. By screening Sequence Read Archive, additional genes encoding 16S rRNA and alkyl/benzyl-succinate synthases analogous to those in Hermodarchaeota were identified in metagenomic datasets from a wide range of marine and freshwater sediments. These findings suggest that Asgard archaea capable of degrading alkanes and aromatics via formation of alkyl/benzyl-substituted succinates are ubiquitous in sediments.

## Introduction

Alkanes and aromatic hydrocarbons are abundant and prevalent in the environment. Although they are major components of petroleum, living organisms are also their important sources. These compounds are inactive in molecular structure, which makes them relatively inert substrates. Under aerobic conditions, alkanes can be oxidized by monooxygenase or dioxygenase in aerobic microorganisms, which use oxygen to supply a reactive oxygen species. The resulting alcohols are further oxidized to aldehydes by dehydrogenases, which are then converted to fatty acids [1]. For the anaerobic oxidation of alkanes, several mechanisms have been found in anaerobic microorganisms. Sulfate-reducing bacteria (SRB) are capable of activating n-hexadecane [2], propane, and n-butane [3] through the addition to fumarate, producing alkyl-substituted succinates. This is analogous to the anaerobic activation of aromatic hydrocarbons, yielding benzylsuccinate as the first intermediate, in the denitrifier *Thauera aromatica* [4]. For Archaea, only a few anaerobic species are currently known to have the ability to grow on hydrocarbons, with the exception of methane. *Ferroglobus placidus* can degrade benzene at 85 °C, coupling reduction of Fe(III) [5]. A thermophilic sulfate-reducing archaeon, *Archaeoglubus fulgidus*, is found to be able to oxidize long-chain n-alkanes anaerobically. It is inferred that *Archaeoglubus fulgidus* may activate alkane through binding to fumarate, which is catalyzed by alkylsuccinate synthase [6]. Recently, a thermophilic archaea (*Candidatus Syntrophoarchaeum*) is shown to activate butane via alkyl-coenzyme M formation under anaerobic conditions, which is similar to anaerobic activation of methane [7]. Genes encoding a similar methyl-coenzyme M reductase (MCR) complex have also been identified in genomes of uncultivated Bathyarchaeota [8], Hadesarchaeota [9] and Helarchaeota [10].

The recently discovered Asgard superphylum is a group of archaea with many eukaryotic features including six distinct phyla: Loki-, Thor-, Odin-, Heimda-, Hel-, and Gerd-archaeota [10–12]. These archaea possess so-called eukaryotic signature proteins (ESP), which, in eukaryotes, are involved in membrane-trafficking processes, vesicle biogenesis and trafficking, cytoskeleton formation and remodeling, endosomal sorting complexes required for transport-mediated protein degradation, and endosomal sorting; therefore, they are deemed representative of the closest archaeal relatives of eukaryotes [11]. Diversity investigations have revealed that Asgard archaea are widely distributed in various anoxic environments, including mangrove sediments, estuarine sediments, freshwater sediments, hydrothermal habitats, marine sediments, cold seeps, hot springs, mud volcanos, and soils [10, 11, 13, 14]. Genomic analysis suggests that Asgard archaea may primarily be organoheterotrophs but some of them, such as Lokiarchaeota, Thorarchaeota and Gerdarchaeota, may also be mixotrophs, which can perform carbon fixation via the Wood–Ljungdahl pathway (WLP) [11, 12, 14]. The versatile lifestyles of Lokiarchaeota, Thorarchaeota and Gerdarchaeota have been supported by metatranscriptomics [12, 14]. More recently, Helarchaeota from hydrothermal deep-sea sediments is suggested to have the potential to oxidize short-chain hydrocarbon using MCR-like enzymes [10], similar to the butane-degrading archaea *C. Syntrophoarchaeum*; Lokiarchaeota from deep Costa Rica sediments is found to contain genes encoding glycyl-radical enzyme and benzoyl-CoA reductase, suggesting their potential ability to degrade alkanes or aromatic hydrocarbons [15]. These results underscore the roles of Asgard archaea in global carbon cycling.

Here, we present the discovery of metagenome-assembled genomes (MAGs) recovered from anoxic mangrove sediment belonging to a new Asgard phylum that has the potential to carry out anaerobic oxidation of alkanes and aromatic compounds through binding to fumarate producing alkyl/benzyl-substituted succinates, which further extends our knowledge on carbon metabolism of Asgard archaea.

## Methods

### Sample collection and metagenomic sequencing

Six sediment samples were taken from the site H0 at mangrove swamps on Techeng Island, Guangdong, China on November 25, 2018 (Supplementary Fig. 1). The site was located in a 500-year-old mangrove wetland in Zhanjiang national nature reserve. Two sediment cores with 1 m depth (H02 and H03) were collected using a peat sampler and they were ~1 m apart. The fresh cores were divided into surface (15–20 cm), intermediate (40–45 cm), and bottom (95–100 cm) sections in an anoxic glove box, and stored at −80 °C for analyses of sediment physiochemical characteristics and microbes. Detailed sediment properties are provided in Supplementary Table 1.

Genomic DNA was obtained from about 5–10 g of sediment samples with PowerSoil DNA Isolation Kit (MoBio Laboratories, Carlsbad, CA, USA). The DNA concentration was determined with Qubit 2.0 and Nanodrop One (Thermo Fisher Scientific, Waltham, USA). About 10–20 μg of DNA was retrieved from each sediment sample. Sequencing libraries were prepared using NEB Next Ultra DNA Library Prep Kit (New England Biolabs, MA, USA) following manufacturer’s protocols. Metagenomic sequence data for the six sediment samples was produced using Illumina HiSeq 2500 instruments at Guangdong MagiGene Technology Corporation (Guangzhou, China). The amount of raw sequence data was approximately 60 Gbp for each sample (2 × 150 bp).

### Metagenome assembly and genome reconstruction

Raw reads were trimmed to get rid of adapters and low-quality reads with Trimmomatic [16]. The high-quality metagenomic sequences were assembled using either MEGAHIT [17] with the following parameters: --k-min 27--k-max 127--k-step 10, or IDBA-UD [18] using the parameters: -mink 55 -max-k 105 -steps 10. Genome binning was performed using an association of MetaBAT [19], Emergent Self-Organizing map (ESOM) [20], MaxBin [21], and CONCOCT [22]. Briefly, high-quality reads from each sample were mapped to the assembled contigs of the sample using Bowtie2 [23]. The resulting sam file was processed to bam file with samtools [24], which was applied to compute coverage information of contigs using jgi_summarize_bam_contig_depth in MetaBAT [19]. Genomic bins were produced by MetBAT [19], MaxBin [21], and CONCOCT [22] based on the obtained coverage information. In parameter setting of the three algorithms, contigs with length <1500 bp in each assembly were filtered. Outlier contigs/scaffolds in bins was checked carefully and removed using ESOM [20] and mmgenome toolbox (http://madsalbertsen.github.io/mmgenome/). For ESOM [20], these bins were sheared into short fragments (5–10 kb) and grouped according to the tetranucleotide frequency in ESOM [20] (Supplementary Fig. 2). Subsequently, the resulting groups were sorted manually and outlier fragments were excluded. The bins were recovered with getClassFasta.pl. Two control genomes (*Methanosarchina mazei* strain Goe1 and *Methanosphaera stadtmanae*) were added to raise ratio of signal to noise and improve binning (Supplementary Fig. 2); they were imported into ESOM [20] together with the bins we obtained. Sequence fragments produced by the control genomes had a sharp boundary, which made manual curation easier; the extracted control genomes were compared with original genomes, and complete recovery was possible. The GC content of contigs in each bin was computed using mmgenome R package (https://github.com/MadsAlbertsen/multi-metagenome/blob/master/R.data.generation/calc.gc.pl). The GC content and coverage information for contigs of the bins were used to draw a scatter plot in which the clustered spots representing one single bin were detected with the mmgenome R package and discrete spots were precluded. Finally, the bins obtained were curated manually to further remove outlier contigs/scaffolds based on multi-copy marker genes. CheckM [25] was used to assess completeness and contamination of these genome bins. As a result, seven high-quality genome bins generated using the MEGAHIT [17] assembly, belonging to a unknown phylum of Asgard archaea, were sorted for further analysis (Supplementary Table 2). These bins were named Hermodarchaeota. Statistical analysis of contigs comprising Hermodarchaeota MAGs was performed using RefineM [26]. The AAI calculated by CompareM (https://github.com/dparks1134/CompareM) was applied to compare the discrepancies between Hermodarchaeota bins and known Asgard genomes.

### 16S rRNA gene analysis

Among the above-mentioned seven Hermodarchaeota bins, only h02m_131 contained one 16S rRNA gene sequence (506 bp in length). To find longer 16S rRNA genes, raw reads were mapped to all Asgard bins obtained in this study using Bowtie2 [23] with default parameters. The resulting reads were combined with reads mapping to unbinned contigs. Subsequently, these reads were assembled using metaSPAdes [27] with the parameters: -k 55, 65, 75, 85, 95. The scaffolds produced were binned using procedures as described above. After contamination was removed, we recovered one bin containing a 16S rRNA gene with a length of 1066 bp (h02s_26) (Supplementary Table 3). This bin and h02s_124 are recovered from the same sample and highly similar each other (98.86% AAI), likely representing the same organism. As the h02s_26 had a higher estimated completeness, it was used for further analysis. In order to search for homologs of Hermodarchaeota 16S rRNA gene sequence, raw reads were mapped to the Silva database [28] and then assembled using MATAM [29] to generate a database containing all 16S rRNA gene sequences in the samples. The 16S rRNA gene sequence of h02s_26 was used to identify homologs within the database using Blastn [30]. Two additional 16S rRNA gene sequences were detected, which had >95% identity with the query sequence (Supplementary Table 3). Furthermore, comparison to the existing 16S rRNA genes of Asgard archaea was performed with Blastn [30].

To examine microbial community composition in mangrove sediments, full-length 16S rRNA genes were assembled from metagenomic datasets using phyloFlash, classified against the Silva SSU database, and quantitated by BBmap (https://sourceforge.net/projects/bbmap/) in phyloFlash [31].

The 16S rRNA genes of Asgard including Hermodarchaeota were aligned along with those of Euryarchaeota with MAFFT-L-INS-I [32] and pruned using trimAl [33]. These Asgard 16S rRNA genes without genomic information are derived from a previous study [12]. Maximum-likelihood (ML) tree was conducted with IQtree [34] (v.1.6.12) using the GTR + F + I + G4 model with 1000 ultrafast bootstraps.

### Phylogenomic analysis of concatenated ribosomal proteins

A suit of representative Archaeal taxa consisting of 361 genomes were used in the analysis; they covered 16 archaeal phyla and included all good-quality Asgard genomes (completeness of >70% and contamination of <10%) available in NCBI database. Genes for all Hermodarchaeota bins and reference archaeal genomes were analyzed with Prodigal [35] (v.2.6.3) using the setting: -p meta. Prokka [36] was also used for performing gene prediction but fewer genes were obtained by Prokka [36] than Prodigal [35]. The homologs of 56 ribosomal proteins in seven Hermodarchaeota bins and representative archaeal reference taxa were identified through Archaeal Cluster of Orthologous Groups (arCOGs) using Blastp [37] (cutoff: e-value, 1e−5). To establish a robust alignment, the 56 ribosomal proteins were selected based on the taxa dataset. It was insured that the number of the ribosomal protein genes lost in a genome was minimal. Subsequently, the taxa dataset would be further sorted out to ensure that the remaining taxa contained at least 50 out of 56 ribosomal proteins (~90%). The full lists of organisms and ribosomal proteins used for phylogenomic inference are shown in Supplementary Tables 4 and 5, respectively. Each of the 56 ribosomal proteins was aligned using MAFFT-L-INS-I [32], checked manually, and pruned using trimAl [33] under automated 1 or BMGE [38] with BLOSUM30 matrix. Trimmed alignments were concatenated, and then further aligned with MAFFT-L-INS-I [32] and pruned using BMGE [38] or trimAl [33] to generate a final alignment. An ML phylogeny was inferred with IQtree [34] using the LG + C60 + F + G + PMSF model and ultrafast bootstrapping (1000 replicates).

### Annotation of genome bins and metabolic reconstruction

Gene annotation was done by searching against the non-redundant (nr) database (downloaded from the National Center for Biotechnology Information (NCBI) in October 2019), existing arCOGs [39], and the UniProtKB and Swiss-Prot database [40] using Blastp [37] (cutoff: e-value, 1e−5). In addition, functional annotation for coding sequences was also conducted using eggNOG-mapper [41] against the eggNOG database. To reconstruct the metabolic pathways, annotation of coding sequences was performed on the BlastKOALA server [42]. Protein conserved domains were analyzed using InterProScan [43] with default parameters. Carbohydrate-active enzyme annotation was conducted on the dbCAN2 meta server [44]. MEROPS database [45] was used to analyze peptidases of Hermodarchaeota genomes.

To identify genes related to hydrocarbon utilization in Hermodarchaeota bins, a protein database was constructed comprising enzymes involved in anaerobic hydrocarbon oxidation in the nr database. These enzymes included the α-subunit of alkylsuccinate synthase (AssA), α-subunit of the benzylsuccinate synthase (BssA), pyruvate formate lyase (pfl), pyruvate formate lyase-activating enzyme, glycyl radical enzyme, (1-methylalkyl) succinate synthase (Mas), and α-subunit of naphtylmethylsuccinate synthase (Nms). Hermodarchaeota genes were queried against this database using Blastp [37] (cutoff: e-value, 1e−5). Positive hits were compared with the annotations from UniProt [40], Swiss-Prot, and Interpro [46]. Motifs or conserved residues for AssA/BssA and Ass/Bss-activating proteins were analyzed by comparing these hits with known reference proteins following a previous method [6]. Conformation of AssA/BssA was analyzed with I-TASSER v. 5.1 [47] using the default setting. The *bzd* gene cluster in the contig with benzoyl-CoA reductase (Bcr) was analyzed according to an anaerobic benzoate degradation pathway reported previously [48].

Nitrate reductases from the nr database were used for the construction of a protein database. The predicted genes in Hermodarchaeota bins were applied to search the database by using Blastp [37] (cutoff: e-value, 1e−5). The hits with a bit score >200 were further identified using a hmmscan search against nitrate reductases from TIGRfam [49] and Pfam [50]. The conserved residues and motifs in nitrate reductases were analyzed following a previous method [51]. These hits with the molybdopterin oxidoreductase domain and CXXCXXXC motif were regarded as likely nitrate reductases.

Hydrogenases were identified by searching against a protein database consisting of hydrogenase sequences from nr database using Blastp [37] (cutoff: e-value, 1e−5). Hits were further verified using the HydDB [52], Interpro [46], and KEGG databases [53]. Group 4 NiFe hydrogenases and energy-converting hydrogenases related complexes were identified according to a previous reported method [54]. The CxxC motifs of hydrogenase that link H_2_ metal centers [55] were analyzed.

For identification of ESPs, accession number from the InterPro [46] and arCOGs provided in a ESP table reported by Zaremba-Niedzwiedzka et al. [11] was used for searching for Hermodarchaeota genes annotated by InterPro [46], arCOG [56], and nr databases. The ESPs that were not matched were identified by searching all predicted genes in Hermodarchaeota bins against Asgard homologs using hmmscan.

### Metatranscriptomic analysis

Seven metatranscriptomic datasets from mangrove sediments were obtained from the NCBI database (Accession number: SRR7284884, SRR7284896, SRR7286070, SRR7286715, SRR11241196, SRR11241197, SRR11241198). The sampling locations for these sediments are shown in Supplementary Fig. 1. Transcripts were identified by comparing against a database of genes involved in important metabolic processes from the Hermodarchaeota genomes using Blastn (cutoff: e-value, 1e−10). If a read was mapped to different genes, the best hit was used to determine the number of transcripts assigned to a gene [57]. Among the seven metatranscriptomic datasets, one (SRR11241197) was found to contain more reads matching h02s_68 *Ass/Bss* and *Bcr* genes. Subsequently, this metatranscriptome was assembled using Trinity [58]. The resulting gene fragments were searched against Hermodarchaeota Ass/Bss and Bcr sequences with Blastx [59].)

### Phylogenetic analysis of key functional genes

#### AssA/BssA sequence phylogeny

AssA/BssA sequences from Hermodarchaeota were used as seeds to search against protein sequences of Archaea and Bacteria in the nr database using Blastp [37]. In the BLAST results, sequences with an abnormal size were discarded while protein sequences that have a size ranging from 500 to 1,000 amino acids were retained. For these remaining sequences, the top 1000 homologs with an E-value <1e−132 and total score >415 were selected to construct a dataset. Furthermore, these sequences in this dataset were clustered using CD-HIT [60] (≥90% identity), and the repeated sequences with >90% amino acid identity were removed. Finally, 281 homologs were retrieved from Bacteria and 18 homologs from Archaea. These sequences, along with AssA/BssA sequences from Hermodarchaeota and other Asgard lineages, were arrayed with MAFFT-L-INS-I [32], and then pruned using BMGE [38] (BLOSUM30 option). An ML phylogenic tree was computed with IQtree [34] (v.1.6.12) using the LG + I + G4 model and ultrafast bootstrapping (1000 replicates).

#### Nitrate reductase sequence phylogeny

Nitrate reductases of prokaryotes are classified into two major clusters, the membrane-associated prokaryotic nitrate reductase (Nar) cluster, and the periplasmic nitrate reductase (Nap)/prokaryotic assimilatory nitrate reductase (Nas) cluster. To determine evolution of nitrate reductases from Hermodarchaeota, a set of homologs of Nar, Nap, and Nas from representative prokaryotic organisms were used for the reconstruction of a phylogenetic tree according to a previous study [51]. In addition, nitrate reductase sequences from Hermodarchaeota were searched against protein sequences in the nr database using Blastp [37]; the top 100 hits were also included in the phylogenetic tree. These sequences were arrayed with MAFFT-L-INS-I [32] and pruned using BMGE [38] (BLOSUM30 option). An ML phylogeny was inferred with IQtree [34] using the best parameters obtained by internal prediction model (LG + F + I + G4), and ultrafast bootstrapping (1000 replicates).

#### Bcr sequence phylogeny

Bcr sequences from Hermodarchaeota were searched against the nr database using Blastp [37]. In the BLAST results, homologs with an E-value <2.86 e−36 were sorted. These sequences were filtered using CD-HIT [60] (≥90% identity) to reduce the amount of sequences. In the resulting sequences, only sequences annotated as Bcr were used for constructing the phylogeny. These sequences were arrayed using MAFFT-L-INS-I [32] and pruned using BMGE [38] (BLOSUM30 option). An ML phylogenic tree was inferred with IQtree [34] using the best parameters obtained by internal prediction model (*bcrN*: LG + G4; *bcrO*: LG + I + G4; *bcrQ*: LG + I + G4), and ultrafast bootstrapping (1000 replicates).

#### Reductive dehalogenase sequence phylogeny

Functionally characterized reductive dehalogenases from bacteria have been reported previously [61]. The sequences, together with sequences of reductive dehalogenase from Hermodarchaeota and other Asgard lineages, were arrayed using MAFFT-L-INS-I [32] and pruned using BMGE [38] (BLOSUM30 option). An ML phylogenic tree was inferred with IQtree [34] using the best parameters obtained by internal prediction model (LG + I + G4), and ultrafast bootstrapping (1000 replicates).

#### [NiFe]-hydrogenase (large subunit) sequence phylogeny

Group 3 and group 4 [NiFe]-hydrogenases from Hermodarchaeota identified by HydDB [52] were filtered by length and motifs, and only sequences with >300 amino acids and CxxC motifs were used. Reference hydrogenases were downloaded from HydDB [52] and repeated sequences were removed by cd-hit. Sequences of hydrogenase large subunit from other Asgard archaea were derived from a previous study [62]. All hydrogenase sequences were arrayed with MAFFT-L-INS-I [32] and pruned using BMGE [38] (BLOSUM30 option). An ML phylogenic tree was performed with IQtree [34] using the predicted best model (LG + I + G4), and ultrafast bootstrapping (1000 replicates).

### Environmental distribution of Hermodarchaeota Ass/Bss and 16S rRNA genes

More than 1000 publicly available environmental metagenomes within the SRA were downloaded from the NCBI website. Reads within these metagenomes were identified by searching against AssA sequences of Hermodarchaeota using the BLASTX of DIAMOND [63] (v.0.9.25) (cutoffs: e-value <1e−5, bit score >50). The Hermodarchaeota Ass/Bss homologs identified are shown in Supplementary Table 6.

For identification of 16S rRNA gene fragments belonging to the Hermodarchaeota, reads in metagenomes from the SRA database were mapped to the 16S rRNA sequence of the h02s_26 genome using BWA [64] with default parameters. CoverM (v.0.3.1) (https://github.com/wwood/CoverM) was applied to filter reads. These reads were regarded as matched appropriately if the alignment length was ≥50% and the identity was ≥90%.

## Results and discussion

### Identification of Hermodarchaeota genomes from mangrove swamps

**Fig. 1:**
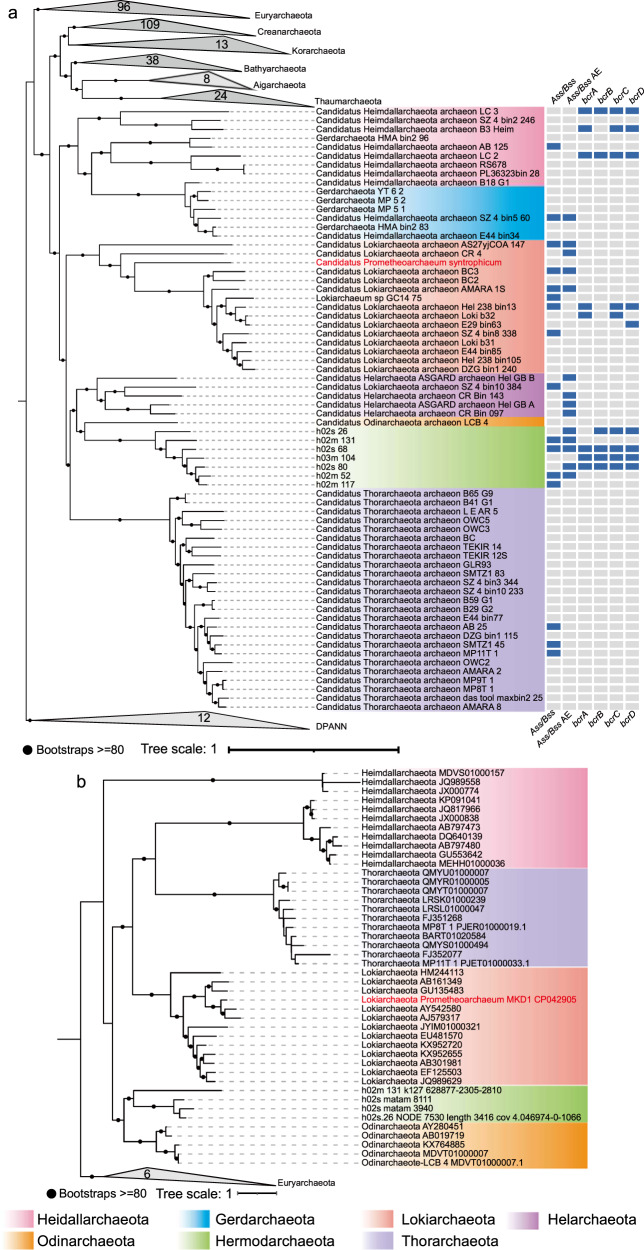
Phylogenetic placement of Hermodarchaeota within the Asgard archaea superphylum. **a** Phylogenomic tree of 56 concatenated ribosomal proteins reconstructed using IQtree with the LG + C60 + F + G + PMSF model. Nodes with ultrafast bootstrap values ≥80 are indicated by black circles. The distribution of key genes involved in degradation of hydrocarbons in Asgard archaeal genomes is also presented. Ass/Bss alkyl/benzyl-succinate synthase gene, Ass/Bss AE alkyl/benzyl-succinate synthase activating enzyme gene, bcrABCD benzoyl-CoA reductase ABCD subunit genes. **b** Maximum-likelihood phylogenetic tree of 16S rRNA gene sequences belonging to Hermodarchaeota were inferred using IQtree with GTR + F + I + G4 model. Black circles indicate bootstrap values ≥80.

A total of 360 gigabases of raw sequence data were obtained from above-mentioned six sediment samples (Supplementary Fig. 1 and Supplementary Table 1). Metagenomic de novo assembly and binning generated the reconstruction of over 112 archaeal genomes (>50% complete). Among them, 22 genomes belonged to the Asgard superphylum. Partial high-quality Asgard genomes are shown in Supplementary Table 2. Maximum likelihood phylogenic trees were reconstructed using 56 concatenated ribosomal proteins or 122 concatenated archaeal-specific marker proteins. The phylogenetic analyses revealed seven MAGs representing a novel group of the Asgard archaea (Fig. 1a and Supplementary Fig. 3). These MAGs are located in distinct lineages and form a distantly related cluster with the Odinarchaeota in a phylogenetic tree with high bootstrap support (Fig. 1a). They had a bin size ranging from 1.86 to 5.10 Mbp and a 43.1–48.7% mean GC content (Table 1**)**. Genome completeness of the MAGs ranged from 74.7% to 92.7% and there was almost no contamination of other genome fragments detected. These MAGs were recovered from top-layer (0.15–0.2 m) and mid-layer (0.4–0.45 m) samples of mangrove swamp sediment.

**Table 1:** Genomic features of Hermodarchaeota bins.

Bin ID	h02s_80	h02m_131	h02s_68	h02m_52	h02m_117	h03m_104	h02s_26
Completeness (%)	92.67	89.5	86.22	77.46	74.69	78.04	76.42
Contamination (%)	0	1.87	0.47	0	0	0	1.94
Strain heterogeneity (%)	0	0	0	0	0	0	0
Number of coding genes	3636	3895	4833	2582	1785	2561	2508
GC content (%)	44.54	43.21	43.91	44.52	44.71	43.05	48.74
Contig/scaffold number	267	863	903	357	269	469	677
Estimated genome size (Mbp)	3.76	4.22	5.10	2.68	1.86	2.66	2.53
N50 (bp)	20636	6335	6054	11896	8731	6995	4119
Longest contig/scaffold (bp)	99342	54136	46141	68536	36381	32924	28150

Genome completeness, contamination, and heterogeneity were estimated using CheckM^43^.

Two partial 16S rRNA genes (1066 bp and 506 bp in length) were identified from MAGs of h02s_26 and h02m_131, respectively (Supplementary Table 3). By blasting against all 16S rRNA gene sequences from the six samples, two additional 16S rRNA genes were found to have more than 95% identity with that of h02s_26 (Supplementary Tables 3 and 7). Phylogenetic analyses revealed that the four 16S rRNA gene sequences formed a phylogenetically distinct group from the Odinarchaeota, and their position was similar to that of the above-mentioned seven MAGs in the genomic trees inferred by concatenated marker proteins (Fig. 1b). The 16S rRNA gene sequences of h02s_26 and h02m_131 showed a phylum level divergence with a DNA identity of 72.9–83.7% [65] when compared with other Asgard archaeal 16S rRNA gene sequences (83.7% and 79.9% sequence similarity to that of Odinarchaeota, respectively) (Supplementary Table 7). Here, we propose *Candidatus* “Hermodarchaeota” as the name of this new group, after Hermod, the son of the god Odin in Norse mythology. An analysis of the average amino acid identity (AAI) revealed that these genomes have an AAI of 41.12–47.48% to other Asgard archaea (Supplementary Fig. 4) and fall within the range (40–52%) recommended for the phylum-level classification [66]. This further supports classification of the novel group as a separate phylum in Asgard superphylum. The genomic phylogenetic analysis and AAI suggest that these MAGs represent three different genera within the Hermodarchaeota (Fig. 1a and Supplementary Fig. 3). The MAGs h02m_117, h02s_80, h02m_52, h02s_68 and h03m_104 represent one genus, h02m_131 represents the second, and h02_124 represents the third. In addition, Hermodarchaeota MAGs possessed a suit of eukaryotic signature proteins (ESPs) that have been identified in other Asgard archaea (Supplementary Table 8).

### Metabolic reconstruction of Hermodarchaeota

**Fig. 2:**
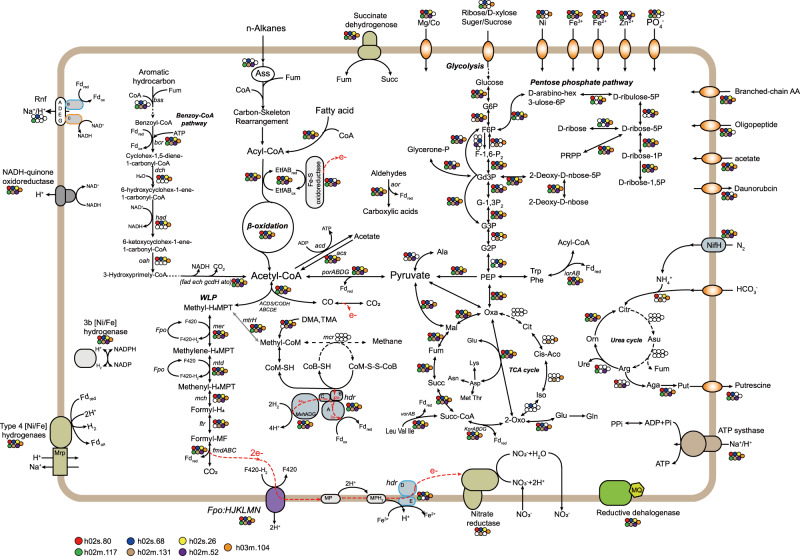
Key metabolic pathways in Hermodarchaeota genomes. The presence or absence of enzymes in these pathways is indicated with dots with different colors. If the dot corresponding to a genome is colorless, it indicates that the enzyme is absent in the genome. Red dotted lines represent electron flow. Genes related to the pathways presented in this figure are given in Supplementary Table 9. WLP Wood–Ljungdahl pathway, TCA tricarboxylic acid, DMA dimethylamine, TMA trimethylamine, MP methanophenazine, Fd ferredoxin.

Similar to Lokiarchaeota and Thorarchaeota [62], metabolic analysis of Hermodarchaeota uncovered the presence of genes involved in the complete WLP (Fig. 2, Supplementary Table 9). The WLP is traditionally connected with methanogenesis in Archaea. However, all Hermodarchaeota genomes lacked genes that encode MCR and genes of key subunits that encode Na^+^-translocating methyl-THMPT:coenzyme M methyltransferase (MTR). Therefore, Hermodarchaeota are unable to perform hydrogenotrophic CO_2_-reducing methanogenesis. Each Hermodarchaeota genome contained three to five copies of gene encoding trimethylamine methyltransferases (*mtt*) and corresponding corrinoid proteins (*mttc*), as well as one to two copies of gene for methylcobamide: CoM methyltransferase (*mtbA*), which is required for methyl-coenzyme M production from trimethylamine [67]. All of the Hermodarchaeota harbored two to four copies of *mtrH* subunit genes, and one to two of them were found to be collocated with a gene encoding corrinoid protein homolog (Supplementary Table 9). The gene operon has been suggested to be involved in transfer of methyl group directly to tetrahydromethanopterin (H_4_MPT) in some methylotrophic methanogens [8, 68, 69]. This may allow the Hermodarchaeota to use methyl compound by establishing a link between methyl-H_4_MPT and methyl-coenzyme M or methyl compound (Fig. 2), as suggested for recently reported Thorarchaeota [62].

**Fig. 3:**
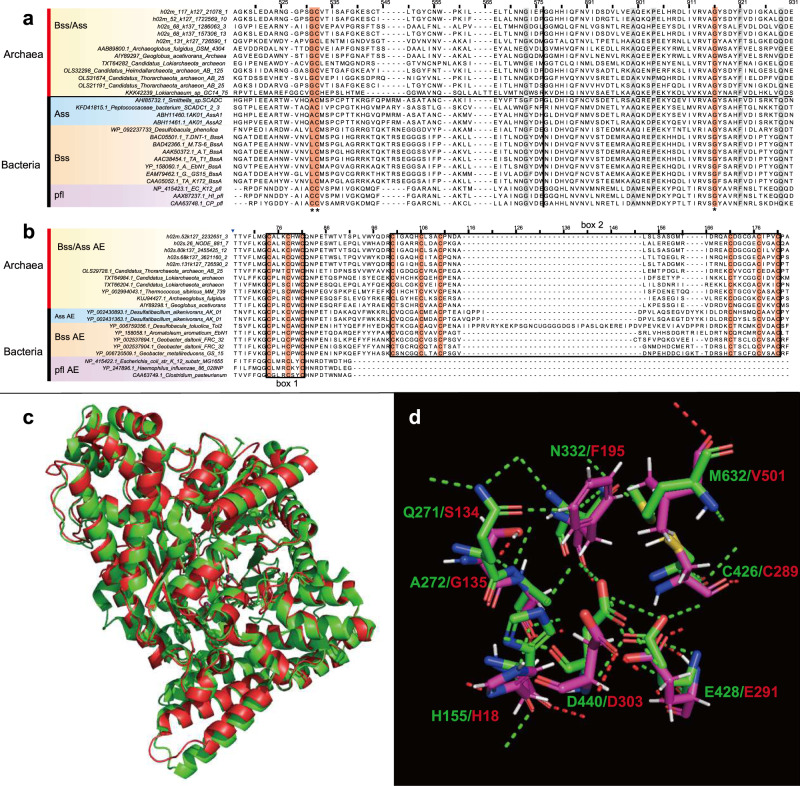
Partial sequence alignment of Hermodarchaeota alkylsuccinate synthase (Ass)/benzylsuccinate synthase (Bss) with known Ass/Bss, and Hermodarchaeota Ass or Bss-activating enzyme (Ass/Bss AE) with known Ass/Bss AE, and structural modeling and active sites of the h02s_68 Ass/Bss. **a** Sequence comparison of Ass/Bss. The regions containing the conserved cysteine residue (**, shaded in red) and the conserved glycine residue (*, shaded in red) are presented. Vertical black line in the middle of sequences represents truncated section. D. A_ AK-01, *D. alkenivorans* strain AK-01. **b** Sequence comparison of Ass/Bss AE. Boxes 1 and 2 correspond to the CxxxCxxC sequence motif and two cysteine-rich regions, respectively, and they are involved in FeS cluster binding. **c** Model of the h02s_68 Ass (red) superimposed on PflD crystal structure (green) (PDB ID: 2f3oA) of *A. fulgidus*. A high alignment confidence (TM-score: 95.6%) was observed between the h02s_68 and *A. fulgidus* crystal structure. **d** Model of the active sites of the h02s_68 Ass (red) overlaid onto PflD (green) of *A. fulgidus*.

**Fig. 4:**
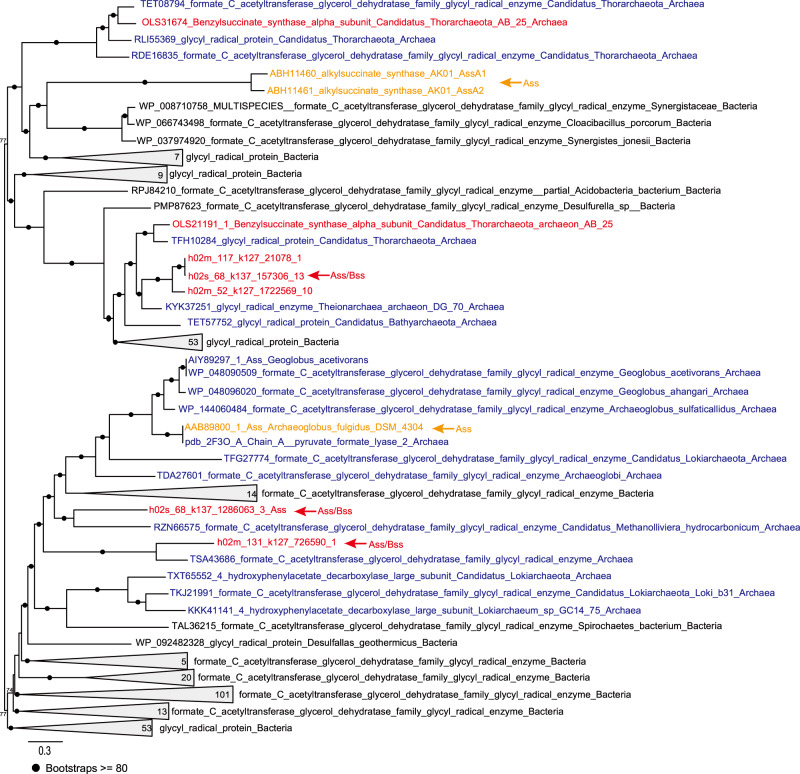
Maximum-likelihood tree of alkyl/benzyl-succinate synthases (Ass/Bss) identified in Hermodarchaeota genomes and homologs from nr database reconstructed using IQtree with LG + I + G4 substitution model. Hermodarchaeota and Thorarchaeota alkyl/benzyl-succinate synthases were red-coded. The verified alkyl-succinate synthases were shaded in yellow. Archaeal homologs were shaded in blue. The Nodes with ultrafast bootstrap values ≥ 80 are indicated by black circles.

In h02m_131, h02s_68, h02m_117 and h02m_52 genomes, five homologs of alkylsuccinate synthase (Ass) or benzylsuccinate synthase (Bss) were identified (Fig. 2, Supplementary Table 9). Their amino acid sequences exhibited 29–33% identity with AssA1 (ABH11460) from *D. alkenivorans* strain AK-01 (bit score: 262–320; e-values: e−98–7e−79) and 29–32% identity with BssA (YP158060) from *A. aromaticum* EbN1 (bit score: 234–294; e-values: 3e−90–e−68), but only had 24–27% identity with the pyruvate formate lyase Pfl (NP415423) from *Escherichia coli* (bit score: 113–165; e-values: 4e−46–3e−29) (Supplementary Table 10). Furthermore, higher similarity was observed between Ass/Bss of Hermodarchaeota and PflD (AAB89800) of *Archaeoglobus fulgidus* (identity: 33–38%; bit score: 336–463). The PflD has been suggested to possess an Ass activity in *A. fulgidus* [6]. The multiple sequence alignment revealed that, similar to other AssA and BssA, the five homologs in Hermodarchaeota harbor only one conserved cysteine which is used to receive the radical from the glycyl residue and initiate the reaction, whereas pyruvate formate lyases, such as Pfl from *E. coli*, possess two neighboring conserved cysteines in the region [6] (Fig. 3a). Based on the 12 known archaeal sequences (Fig. 3a), AssA/BssA in archaea tends to substitute the first cysteine with glycine at the conserved region for PFLs. We analyzed conformation of h02s_68 Ass/Bss using I-TASSER [47]. The displayed structural model was superimposed on PflD crystal structure (PDB ID: 2f3oA) of *A. fulgidus* with high alignment confidence (TM-score: 95.6%) (Fig. 3c). However, among eight active sites reported in the PflD, amino acids of four sites were changed (Fig. 3d). It is unclear if these amino acid changes affect the type of substrates. A phylogenetic analysis was performed using Hermodarchaeota Ass or Bss sequences and their closest homologs (Fig. 4). This revealed that Ass/Bss encoded by Hermodarchaeota are not monophyletic, but intermixed with bacterial sequences, indicating that these Ass/Bss sequences were likely obtained through horizontal gene transfers from bacteria, similar to the pflD of *A. fulgidus* [6]. Specifically, it is inferred that at least two separate horizontal gene transfers occurred between different bacterial donors and members of Hermodarchaeota (Fig. 4).

In addition to *Ass/Bss* genes, among seven Hermodarchaeota genomes, five contained one gene encoding the Ass or Bss-activating enzyme (Ass/Bss AE) (Supplementary Table 9), which is needed for Ass/Bss. A Blastp [37] search found that they had greater similarity with AssD2 (YP_002431363) and AssD2’ (YP_002429341) from *D. alkenivorans* strain AK-01, PflC (KUJ94427) of *A. fulgidus*, and BssD (CAA05050) from *T. aromatica* K172, compared to the PflA-activating enzyme (NP_415422) of *E. coli* (Supplementary Table 11). The N-terminal regions of Hermodarchaeota Ass/Bss AEs contained a CxxxCxxC sequence motif (box 1) and two cysteine-rich regions (box 2) (Fig. 3b). Box1 is necessary for the Fe-S cluster of SAM-radical enzymes [70] while box 2 is involved in Fe-S cluster binding, which is unique to Ass/Bss AE and not found in pyruvate formate lyase-activating enzymes [6]. Generally, these data indicate that Hermodarchaeota possess Ass or Bss and activating enzymes. Furthermore, a publicly available metatranscriptome (SRR11241197) from mangrove sediment was assembled. Among the generated gene fragments, two were found to be highly homologous to Hermodarchaeota *Ass/Bss* genes (>60% amino acid identity, >93% alignment length) (Supplementary Table 12), likely suggesting that Hermodarchaeota *Ass/Bss*-like genes may be expressed in mangrove sediment.

Once alkanes are activated, the alkyl-substituted succinates formed will be subjected to thioesterification, carbon-skeleton rearrangement, and decarboxylation [71]. At present, the genes involved in these reactions remain unclear. In *D. alkenivorans* strain AK-01, it is postulated that these steps were catalyzed by acyl-CoA synthetase (ligase) (AMP-forming), methylmalonyl-CoA mutase, and methylmalony-CoA carboxyltransferase [71]. The genes for all of these were present in each Hermodarchaeota genome (Supplementary Table 9). Subsequently, acyl-CoA produced from alkane oxidation can be oxidized to acetyl-CoA by related enzymes of the beta-oxidation including acyl-CoA dehydrogenase (Acd), enoyl-CoA hydratase (Ech), 3-hydroxyacyl-CoA dehydrogenase (Hadh), and acetyl-CoA acyltransferase (Fad), and the genes encoding these enzymes have been identified in Hermodarchaeota genomes (Supplementary Table 9). In addition, each Hermodarchaeota genome contained genes for 10–19 Acds, two to three electron transfer flavoprotein complexes (ETF), and one to four FeS oxidoreductases (Supplementary Table 9). This could produce reduced ferredoxin or NADH by electron bifurcation in the ACD/ETF complex for anabolism (Fig. 2) [72, 73]. The genes for acetyl-CoA decarbonylase/synthase:CO dehydrogenase complex (ACDS/CODH) were identified in Hermodarchaeota (Supplementary Table 9); these are key enzymes in the metabolism of acetyl-CoA from beta-oxidation. This suggests that acetyl-CoA can be further oxidized into CO_2_ and yield reduced ferredoxin via the oxidative WLP as previously shown for butane oxidation in *Ca. Syntrophoarchaeum* [7] (Fig. 2).

**Fig. 5:**
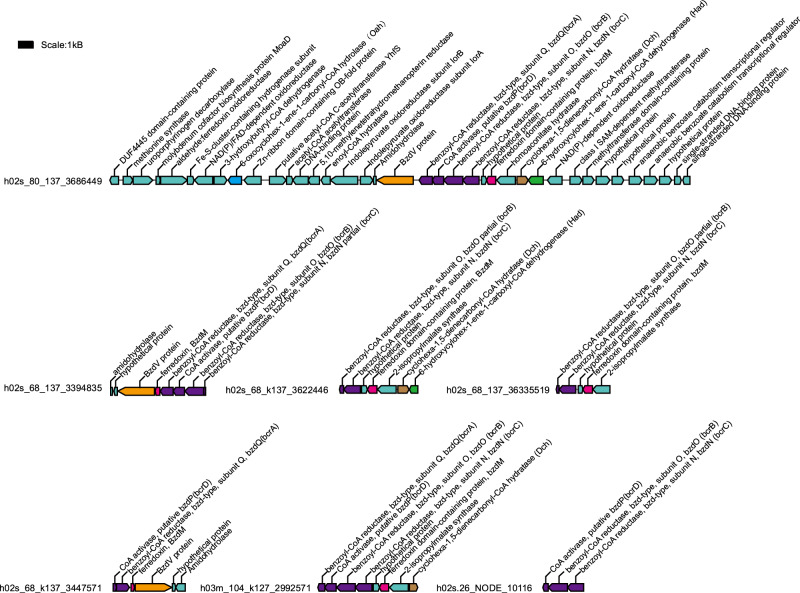
The gene composition in the contigs containing the benzoyl-CoA reductase operon in Hermodarchaeota genomes. Purple bocks indicate gene operon for the benzoyl-CoA reductase. Arrows show transcriptional orientation of the genes.

For anaerobic oxidation of aromatic hydrocarbons, the first intermediate formed by the addition of fumarate, benzylsuccinate, is further oxidized to benzoyl-CoA, which is regarded as a primary aromatic intermediate in the anaerobic oxidation of plentiful aromatic hydrocarbons [74]. Hermodarchaeota genomes contained almost all genes found in the benzoyl-CoA pathway (Fig. 2; Supplementary Table 9). Next in this pathway, the conversion from benzoyl-CoA to 3-hydroxypimelyl-CoA, is catalyzed by four key enzymes in *T. aromatica* including benzoyl-CoA reductase (Bcr), cyclohexa-1,5-dienecarbonyl-CoA hydratase (Dch), 6-hydroxycylohex-1-ene-1-carboxyl-CoA dehydrogenase (Had), and 6-oxocyclohex-1-ene-1-carbonyl-CoA hydrolase (Oah) [75], of which all genes were identified in h02s_80, h02s_68, h03m_104 and h02s_26 genomes (Supplementary Table 9). In addition, the genes encoding BcrABCD subunits in these genomes were found collocated in a contig (Fig. 5), forming a gene cluster; in h02s_80, the contig also contained genes for an BzdV protein, a ferredoxin, a Dch, a Had, a NADP-dependent oxidoreductase, and two anaerobic benzoate catabolism transcriptional regulators (Fig. 5). The arrangement of the gene cluster for benzoate catabolism was analogous to that in *T. aromatica* [48]. This indicates that ferredoxin probably acts as electron donor for the reduction of benzoyl-CoA in Hermodarchaeota while NADP-dependent oxidoreductase regenerates reduced ferredoxin as previously shown for *Azoarcus evansii* [76]. Subsequently, 3-hydroxypimelyl-CoA will be further oxidized to acetyl-CoA by the related enzymes via beta-oxidation including Hadh, Fad, glutaryl-CoA dehydrogenase (GcdH), glutaconyl-CoA decarboxylase (GcdA), Ech, 3-hydroxybutyryl-CoA dehydrogenase (PaaH), and acetyl-CoA acetyltransferase (AtoB). The genes for all these enzymes have been detected in Hermodarchaeota (Fig. 2; Supplementary Table 9). In gene fragments assembled from the above-mentioned metatranscriptome, two genes had high similarity to Hermodarchaeota *bcrAC* genes (>60% amino acid identity, ≥98% alignment length) (Supplementary Table 12). It is possible that Hermodarchaeota *bcr-*like genes can be transcribed in mangrove sediment. Collectively, the data suggest that Hermodarchaeota may be capable of using various aromatic hydrocarbons or alkanes as carbon and energy sources.

To examine the distribution of the genes for Ass/Bss, Ass/Bss AE and Bcr in Asgard archaea, we analyzed all known Asgard genomes in nr database (Fig. 1a). Among 118 genomes, nine contained the genes encoding both Ass/Bss and Ass/Bss AE, including one Heimdallarchaeota, five Lokiarchaeota, and three Hermodarchaeota; ten contained the genes for Bcr, including three Heimdallarchaeota, three Lokiarchaeota and four Hermodarchaeota; one (h02s_68 of Hermodarchaeota) harbored all the genes encoding the three enzymes. Statistical analysis of contigs comprising Hermodarchaeota MAGs revealed that the contigs containing genes for Ass/Bss, Ass/Bss AE, Bcr and the Wood–Ljungdahl pathway fell inside the 95^th^ percentile of a typical genome, supporting that these genes do belong to their respective genomes (Supplementary Figs. 5 and 6). These observations highlighted the important roles of members of Hermodarchaeota in the anaerobic degradation of alkanes and aromatic hydrocarbons. Some members of Lokiarchaeota and Heimdallarchaeota may also have the potential capability to anaerobically perform degradation of aromatics. Two Lokiarchaeota MAGs recovered from deep Costa Rica sediments were suggested to possibly utilize benzoate coupling with reduction of nitrate, nitrite and sulfite [15].

Compared to other Asgard archaea [62], Hermodarchaeota genomes possess more sophisticated energy-conserving complexes, including seven subunits of the F_420_H_2_ dehydrogenase (encoded by *fpo*), 11 subunits of NADH-quinone oxidoreductase, flavoprotein and FeS subunit of succinate dehydrogenase/fumarate reductase, group 4 [NiFe]-hydrogenase, the B and C subunits of the Rnf complex, and V/A-type adenosine triphosphate (ATP) synthase (Fig. 2; Supplementary Fig. 7; Supplementary Table 9). Group 4 [NiFe]-hydrogenase was only identified in Heimdallarchaeota and Odinarchaeota while the F_420_H_2_ dehydrogenase and Rnf complex were not found in other Asgard archaea [62]. The presence of group 3b and group 3c [NiFe]-hydrogenases (Supplementary Fig. 7), together with the WLP suggest that Hermodarchaeota may be able to reduce CO_2_ via the WLP using H_2_ as electron donor. The membrane-bound Group 4 [NiFe]-hydrogenase [77], H^+^-translocating F_420_H_2_ dehydrogenase [78], and Na^+^/ H^+^-translocating Rnf complex [79] can couple H_2_ oxidation with Na^+^/ H^+^ translocation across cytoplasmic membrane and further generate an electrochemical ion gradient that drives ATP synthesis. The coupling is absent in other Asgard phyla [62]. Of note, one to two copies of genes that encode D and E subunits of CoB-CoM heterodisulfide reductase (Hdr) were identified in h02s_80, h02s_68, h02m_117 and h02m_52 genomes (Fig. 2), which are absent in most methanogens and other Asgard archaea, but found solely in the *Methanosarcinales* [62, 80]. HdrDE is an integral membrane complex, it accepts electrons from reduced methanophenazine (MPH_2_), and assists in the production of a chemiosmotic gradient across the cell membrane (Fig. 2). In methanogens, the membrane-bound electron transport chain is more efficient than electron bifurcation [81]. It may be speculated that Hermodarchaeota have a higher growth yield than other Asgard archaea without HdrDE complex. In addition, *hdrABC* and *mvhADG* for methyl-viologen-reducing hydrogenase were also detected (Fig. 2), suggesting that the complex possibly leads to co-reduction of CoM-S-S-CoB heterodisulfide and ferredoxin by electron bifurcation mechanism as shown previously for *Methanosarcina acetivorans* [80]. Owing to an absence of MCR, the reaction forming a heterodisulfide of CoM and CoB by MCR is not present in Hermodarchaeota. Although Hermodarchaeota contained genes for cytoplasmic fumarate reductase (Supplementary Table 13), which has been shown to reduce fumarate using CoM-S-H and CoB-S-H in *Methanobacterium thermoautotrophicum* [82], the reaction is not accompanied by energy conservation. Thereby, like most Asgard archaea, it remains to be determined whether the cycle of CoM-S-S-CoB is operative in Hermodarchaeota.

In addition to metabolic pathways for aromatic hydrocarbons and alkanes, Hermodarchaeota genomes also contain multiple peptidases, aminopeptidases, carboxypeptidases, and amino acid and oligopeptide transporters (Supplementary Fig. 8a) that have been identified in other Asgard archaea [62], suggesting that these microorganisms can utilize peptides and proteins as their sources of carbon and nitrogen. Similar to the peptide fermentation of *Pyrococcus furiosus* [83], amino acids hydrolyzed by these peptidases can be oxidatively deaminated by glutamate dehydrogenase (*gdh*), aspartate aminotransferases (*aspC*), 2-oxoglutarate ferredoxin oxidoreductase *(kor*), 2-ketoisovalerate ferredoxin oxidoreductase (*vor*), indolepyruvate ferredoxin oxidoreductase (*ior*), and pyruvate ferredoxin oxidoreductase (*por*) to generate acetyl-CoA and reduced ferredoxin (Fig. 2; Supplementary Table 9). Furthermore, the aliphatic and aromatic aldehydes produced by these oxidoreductases can be oxidized to carboxylic acids by multiple aldehyde ferredoxin oxidoreductases, producing reduced ferredoxin (Fig. 2) [84]. The resulting acetyl-CoA is further converted by acetyl-CoA synthetase to produce acetate (ADP-forming) (Fig. 2), with concomitant formation of ATP [85]. In addition, similar to other Asgard archaea [10, 62], Hermodarchaeota may be capable of using carbohydrates as carbon or energy sources because they possess sugar transporters, sucrose transporters, and various carbohydrate-active enzymes (Fig. 2, Supplementary Fig. 8b). The resulting glucose can then be metabolized to generate intermediates for anabolism via glycolysis and an incomplete citric acid cycle, along with the formation of ATP and reduced nicotinamide adenine dinucleotide (NADH). Subsequently, the NADH is assumed to be oxidized by NADH-quinone oxidoreductase (complex I) to create a transmembrane proton gradient, or be applied to reduce ferredoxin via the Rnf complex (Fig. 2).

Proper electron acceptors are pivotal to successful degradation of aromatic compounds and alkanes. We did not identify any dissimilatory sulfite reductase (Dsr) or anaerobic sulfite reductase (Asr) in Hermodarchaeota genomes. However, Hermodarchaeota genomes contain genes encoding nitrate transporter NrtD and NrtB, as well as homolog of molybdopeterin oxidoreducatase harboring molybdopterin guanine dinucleotide-binding (MGD) domain (Fig. 2; Supplementary Table 9; Supplementary Fig. 9) which is present in nitrate reductase alpha subunit (NarG) [86]. Phylogenetic analysis revealed that these molybdopeterin oxidoreducatases and nitrate reductases from *Desulfovibrio* and Chloroflexi formed a large cluster adjacent to the respiratory membrane-bound Nar clade (Supplementary Fig. 10). Several strains of *Desulfovibrio* have been reported to be capable of performing respiratory nitrate reduction [87, 88]. Furthermore, among these molybdopeterin oxidoreducatase coding genes, two were found to be collocated with a gene encoding 4Fe-4S dicluster domain-containing protein (Supplementary Table 9), a gene arrangement also found in the *E.coli* respiratory *Nar* gene cluster [86]. It is deduced that the molybdopeterin oxidoreducatase and 4Fe-4S dicluster domain-containing protein in Hermodarchaeota may be analogous to NarG and NarH in the membrane-bound nitrate reductase of bacteria. The results suggest that members of Hermodarchaeota may be capable of utilizing nitrate as an electron acceptor during the oxidation of aromatic hydrocarbons as shown previously in denitrifying bacterium *Thauera aromatica* [89]. In addition to nitrate, Fe (III) has been found to serve as the sole electron acceptor of hyperthermophilic archaeon *Ferroglobus placidus* when oxidizing benzoate and phenol. In *Methanosarcina acetivorans*, electrons can be channeled to Fe (III) via HdrDE complex, which drives methane oxidation [90]. Thus, it is possible that Hermodarchaeota can couple oxidation of aromatic hydrocarbons with reduction of Fe (III) through the HdrDE complex (Fig. 2). Similar to Lokiarchaeota and Thorarchaeota, identification of reductive dehalogenase in Hermodarchaeota genomes implicates that these microorganisms are also capable of performing organohalide respiration using chlorinated ethenes/ethanes (Supplementary Fig. 11). We could not identify any pilus and extracellular cytochromes that mediate electron transfer across species, as shown previously in *Ca. S. butanivorans* and ANME archaea [7, 91]. Therefore, it is unclear whether a syntrophic partner organism receiving reducing equivalents exists for members of Hermodarchaeota.

### Environmental distribution

**Fig. 6:**
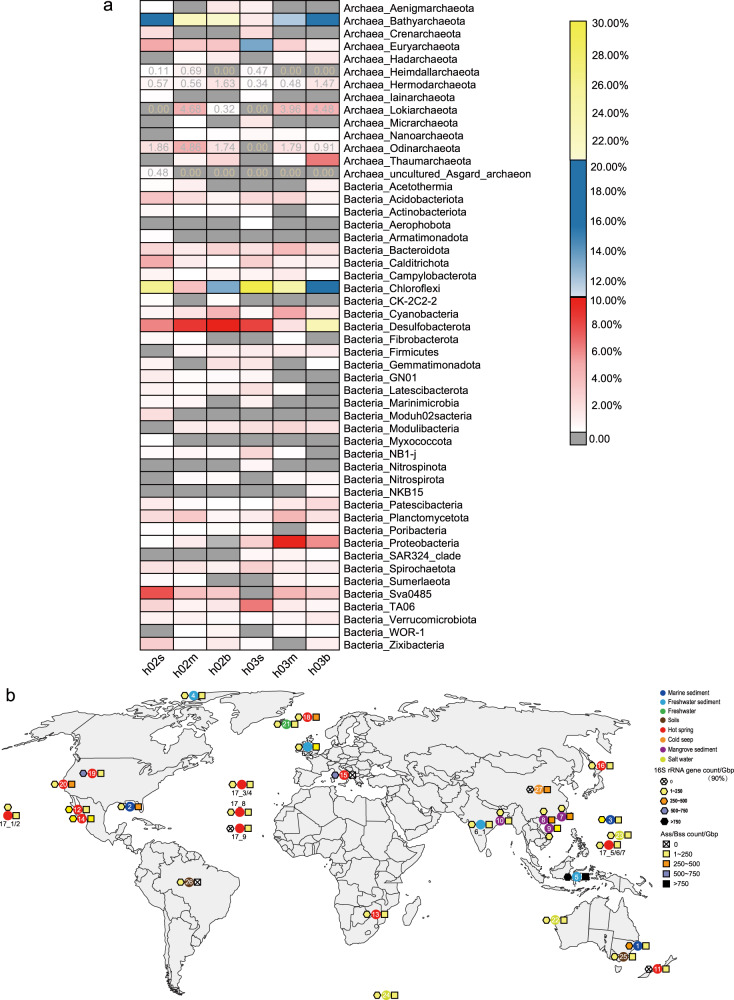
Abundance and distribution of Hermodarchaeota in environments. **a** Microbial community composition from the six mangrove sediments on Techeng Island. The community composition was based on full-length 16S rRNA genes assembled from the metagenomes. **b** Global distribution of homologs of Hermodarchaeota Ass/Bss and 16S rRNA genes identified in metagenomes from various environments. Circles with different colors represent metagenomes from different habitats; the hexagons with different colors indicate the range of 16S rRNA gene count normalized to sequencing depth; the range of Ass/Bss gene count normalized to sequencing depth is indicated using squares with different colors; the numbers in the figure correspond to the ID in Supplementary Table 6.

Based on full-length 16S rRNA genes assembled from the metagenomes using phyloFlash, we analyzed microbial community composition in the six mangrove sediment samples collected from Techeng Island (Fig. 6a). The most abundant phyla were, in a reducing order, Chloroflexi, Bathyarchaeota, Desulfobacterota, Proteobacteria, and Euryarchaeota in these metagenomes. Four Asgard phyla were detected including Hermodarchaeota, Heimdallarchaeota, Lokiarchaeota and Odinarchaeota, with their abundance accounting for 0.81–10.78% of reads matching total full-length 16S rRNA genes in each metagenome. The relative abundance of Hermodarchaeota increased from 0.57% to 1.63% in H02 core with depth while from 0.34% to 1.47% in H03 core with depth. In H02 core, the abundance of Hermodarchaeota appeared to be associated with contents of benzene and methylbenzene in sediments (Supplementary Table 1), possibly further supporting utilization of aromatics by Hermodarchaeota.

To investigate the distribution and diversity of Ass/Bss sequences from archaea, *Ass/Bss* gene sequences from Hermodarchaeota were used to identify homologs in the six mangrove sediment samples in this study and across 1,000 publicly available metagenomes from around the world. A total of 394 nearly full-length *Ass/Bss* genes with archaeal GC motif were recovered from the six mangrove sediment samples (Techeng Island, China) (Supplementary Table 13), likely suggesting that they may be derived from phylogenetically diverse archaea. Only were 34 full-length *Ass/Bss* genes with bacterial AC/LC motif identified from these mangrove sediments (Supplementary Table 13). The results indicate that archaea may be major players in anaerobic degradation of alkanes in some mangrove sediments. Hermodarchaeota *Ass/Bss*-like gene fragments were also detected in various marine and freshwater environments, including marine bay sediments (Fagans, Australia), Guaymas Basin sediments enriched in hydrocarbon seeps (Gulf of California, USA), hot spring sediments (California, USA), deep-sea sediments with petroleum seeps (Eastern Gulf of Mexico), mangrove sediments (Yunxiao, China), Towuti Lake sediments (South Sulawesi, Indonesia), and formation water in coal beds (Qinshui Basin, China) (Fig. 6b, Supplementary Table 6). Furthermore, in these environments, a considerable number of 16S rRNA gene fragments were found to have high sequence similarity (≥90% identity) to that of Hermodarchaeota (Fig. 6b, Supplementary Table 6). Hermodarchaeota 16S rRNA and *Ass/Bss*-like genes were notably in higher relative abundance in the metagenomes generated from deep-sea sediments associated with petroleum seepage, mangrove sediments, Lake Towuti sediments of Indonesia, hydrothermal vent, and hot spring sediments where more hydrocarbons may be present, which was in accordance with attribution of Hermodarchaeota. The homologs of Ass/Bss were also identified in the thermophilic pure archaeon *A. fulgidus* isolated from a submarine hot vent [6] as well as in composite genomes of Thorarchaeota and Lokiarchaeota from deep seabed petroleum seeps [92]. These results suggest that members of Hermodarchaeota, and other archaea capable of performing oxidation of alkanes and aromatic hydrocarbons through addition to fumarate, may be ubiquitous in nature.

In addition to utilization of alkane, Hermodarchaeota is able to perform anaerobic oxidation of aromatic hydrocarbon via addition to fumarate coupling with the benzoyl-CoA degradation pathway. The three subunits of the key enzyme Bcr for the benzoyl-CoA pathway are highly related to those of ATP-consuming class I Bcr of Anaerolineales bacterium of Chloroflexi (Supplementary Fig. 12), suggesting occurrence of horizontal gene transfers between Hermodarchaeota and Chloroflexi. The *bcr* genes and genes for downstream transformation were also identified in composite genomes of Thermoplasmata and Bathyarchaeota from deep-sea sediments with petroleum seeps [92] and Lokiarchaeota from deep Costa Rica sediments [15]. In addition, a new pathway for anaerobic oxidation of short-chain hydrocarbon via alkyl-coenzyme M formation has been proposed in Bathyarchaeota [8], Hadesarchaeota [9], *Ca*. Syntrophoarchaeum [7], and Helarchaeota [10]. These results demonstrate that metabolic processes of hydrocarbons in archaea may be more complicated than thought before. Such complexity likely suggests that utilization of hydrocarbons by archaea may have existed for a long time in the earth. The discovery of Hermodarchaeota and its ubiquitous distribution expands the domain of archaea and has crucial significance for understanding of the ecological functions and evolutionary history of the mysterious Asgard archaea.

## Supplementary information

Supplementary Tables and Figures

Supplementary Table 4

Supplementary Table 6

Supplementary Table 8

Supplementary Table 9

Supplementary Table 13

Dataset 1

Dataset 2

## Data Availability

All sequence data are archived in the NCBI database under BioProject ID PRJNA629047. Genome bins can be found at NCBI under the Accession numbers SAMN14819984 (archaeon H02s_80), SAMN14819985 (archaeon H02S_68), SAMN14819986 (archaeon H02M_131), SAMN14986674 (archaeon H02S_124), SAMN14986675 (archaeon H02M_117), SAMN14986676 (archaeon H02M_52), SAMN14986677 (archaeon H03M_104), and SAMN15489661 (archaeon H02S_26). Raw reads have been submitted to Sequence Read Archive under SRA accession PRJNA629047 with accession numbers SRR11793862, SRR11793863, SRR11793864, SRR11793865 and SRR11793866.

## References

[1] van Beilen JB, Wubbolts MG, Witholt B (1994). Genetics of alkane oxidation by pseudomonas oleovorans. Biodegradation..

[2] Callaghan R, Crowley E, Potter S, Kerr ID (2008). P-glycoprotein: so many ways to turn it on. J Clin Pharmacol..

[3] Joye SB (2007). Anaerobic oxidation of short-chain hydrocarbons by marine sulphate-reducing bacteria. Nature..

[4] Leuthner B, Heider J (1998). A two-component system involved in regulation of anaerobic toluene metabolism in Thauera aromatica. FEMS Microbiol Lett.

[5] Holmes DE, Risso C, Smith JA, Lovley DR (2011). Anaerobic oxidation of benzene by the hyperthermophilic archaeon Ferroglobus placidus. Appl Environ Microbiol..

[6] Khelifi N, Ali OA, Roche P, Grossi V, Brochier-Armanet C, Valette O (2014). Anaerobic oxidation of long-chain n-alkanes by the hyperthermophilic sulfate-reducing archaeon, Archaeoglobus fulgidus. ISME J..

[7] Laso-Pérez R, Wegener G, Knittel K, Widdel F, Harding KJ, Krukenberg V (2016). Thermophilic archaea activate butane via alkyl-coenzyme M formation. Nature..

[8] Evans PN, Parks DH, Chadwick GL, Robbins SJ, Orphan VJ, Golding SD (2015). Methane metabolism in the archaeal phylum Bathyarchaeota revealed by genome-centric metagenomics. Science..

[9] Baker BJ, Saw JH, Lind AE, Lazar CS, Hinrichs K-U, Teske AP (2016). Genomic inference of the metabolism of cosmopolitan subsurface Archaea, Hadesarchaea. Nat Microbiol..

[10] Seitz KW, Dombrowski N, Eme L, Spang A, Lombard J, Sieber JR (2019). Asgard archaea capable of anaerobic hydrocarbon cycling. Nat Commun..

[11] Zaremba-Niedzwiedzka K, Caceres EF, Saw JH, Backstrom D, Juzokaite L, Vancaester E (2017). Asgard archaea illuminate the origin of eukaryotic cellular complexity. Nature..

[12] Cai M, Liu Y, Yin X, Zhou Z, Friedrich MW, Richter-Heitmann T (2020). Diverse Asgard archaea including the novel phylum Gerdarchaeota participate in organic matter degradation. Sci China Life Sci.

[13] Seitz KW, Lazar CS, Hinrichs K-U, Teske AP, Baker BJ (2016). Genomic reconstruction of a novel, deeply branched sediment archaeal phylum with pathways for acetogenesis and sulfur reduction. ISME J..

[14] Cai M, Richter-Heitmann T, Yin X, Huang W-C, Yang Y, Zhang C, et al. Ecological features and global distribution of Asgard archaea. Sci Total Environ. 2020:143581. 10.1016/j.scitotenv.2020.143581. 10.1016/j.scitotenv.2020.143581 33223169

[15] Farag IF, Biddle JF, Zhao R, Martino AJ, House CH, Leon-Zayas RI (2020). Metabolic potentials of archaeal lineages resolved from metagenomes of deep Costa Rica sediments. ISME J.

[16] Bolger AM, Lohse M, Usadel B (2014). Trimmomatic: a flexible trimmer for Illumina sequence data. Bioinformatics..

[17] Li D, Liu C-M, Luo R, Sadakane K, Lam T-W (2015). MEGAHIT: an ultra-fast single-node solution for large and complex metagenomics assembly via succinct de Bruijn graph. Bioinformatics..

[18] Peng Y, Leung HC, Yiu S-M, Chin FY (2012). IDBA-UD: a de novo assembler for single-cell and metagenomic sequencing data with highly uneven depth. Bioinformatics..

[19] Kang DD, Froula J, Egan R, Wang Z (2015). MetaBAT, an efficient tool for accurately reconstructing single genomes from complex microbial communities. PeerJ..

[20] Dick GJ, Andersson AF, Baker BJ, Simmons SL, Thomas BC, Yelton AP (2009). Community-wide analysis of microbial genome sequence signatures. Genome Biol..

[21] Wu Y-W, Simmons BA, Singer SW (2016). MaxBin 2.0: an automated binning algorithm to recover genomes from multiple metagenomic datasets. Bioinformatics..

[22] Alneberg J, Bjarnason BS, De Bruijn I, Schirmer M, Quick J, Ijaz UZ (2014). Binning metagenomic contigs by coverage and composition. Nat Methods..

[23] Langmead B, Salzberg SL (2012). Fast gapped-read alignment with Bowtie 2. Nat Methods..

[24] Li H, Handsaker B, Wysoker A, Fennell T, Ruan J, Homer N (2009). The sequence alignment/map format and SAMtools. Bioinformatics..

[25] Parks DH, Imelfort M, Skennerton CT, Hugenholtz P, Tyson GW (2015). CheckM: assessing the quality of microbial genomes recovered from isolates, single cells, and metagenomes. Genome Res..

[26] Parks DH, Rinke C, Chuvochina M, Chaumeil P-A, Woodcroft BJ, Evans PN (2017). Recovery of nearly 8,000 metagenome-assembled genomes substantially expands the tree of life. Nat Microbiol.

[27] Bankevich A, Nurk S, Antipov D, Gurevich AA, Dvorkin M, Kulikov AS (2012). SPAdes: a new genome assembly algorithm and its applications to single-cell sequencing. J Comput Biol.

[28] Quast C, Pruesse E, Yilmaz P, Gerken J, Schweer T, Yarza P (2012). The SILVA ribosomal RNA gene database project: improved data processing and web-based tools. Nucleic Acids Res..

[29] Pericard P, Dufresne Y, Couderc L, Blanquart S, Touzet H, Birol I (2018). MATAM: reconstruction of phylogenetic marker genes from short sequencing reads in metagenomes. Bioinformatics..

[30] Altschul SF, Gish W, Miller W, Myers EW, Lipman DJ (1990). Basic local alignment search tool. J Mol Biol..

[31] Gruber-Vodicka HR, Seah BK, Pruesse E. phyloFlash–Rapid SSU rRNA profiling and targeted assembly from metagenomes. mSystems. 2020;5:1–16. 10.1128/mSystems.00920-20 PMC7593591 33109753

[32] Katoh K, Standley DM (2013). MAFFT multiple sequence alignment software version 7: improvements in performance and usability. Mol Biol Evol..

[33] Capella-Gutiérrez S, Silla-Martínez JM, Gabaldón T (2009). trimAl: a tool for automated alignment trimming in large-scale phylogenetic analyses. Bioinformatics..

[34] Nguyen L-T, Schmidt HA, Von Haeseler A, Minh BQ (2015). IQ-TREE: a fast and effective stochastic algorithm for estimating maximum-likelihood phylogenies. Mol Biol Evol..

[35] Hyatt D, Chen G-L, LoCascio PF, Land ML, Larimer FW, Hauser LJ (2010). Prodigal: prokaryotic gene recognition and translation initiation site identification. BMC Bioinf..

[36] Seemann T (2014). Prokka: rapid prokaryotic genome annotation. Bioinformatics..

[37] Altschul SF, Madden TL, Schäffer AA, Zhang J, Zhang Z, Miller W (1997). Gapped BLAST and PSI-BLAST: a new generation of protein database search programs. Nucleic Acids Res..

[38] Criscuolo A, Gribaldo S (2010). BMGE (Block Mapping and Gathering with Entropy): a new software for selection of phylogenetic informative regions from multiple sequence alignments. BMC Evol Biol.

[39] Makarova KS, Wolf YI, Koonin EV (2015). Archaeal clusters of orthologous genes (arCOGs): an update and application for analysis of shared features between Thermococcales, Methanococcales, and Methanobacteriales. Life..

[40] Magrane M (2011). UniProt knowledgebase: a hub of integrated protein data. Database.

[41] Huerta-Cepas J, Forslund K, Coelho LP, Szklarczyk D, Jensen LJ, Von Mering C (2017). Fast genome-wide functional annotation through orthology assignment by eggNOG-mapper. Mol Biol Evol.

[42] Kanehisa M, Sato Y, Morishima K (2016). BlastKOALA and GhostKOALA: KEGG tools for functional characterization of genome and metagenome sequences. J Mol Biol..

[43] Jones P, Binns D, Chang H-Y, Fraser M, Li W, McAnulla C (2014). InterProScan 5: genome-scale protein function classification. Bioinformatics..

[44] Yin Y, Mao X, Yang J, Chen X, Mao F, Xu Y (2012). dbCAN: a web resource for automated carbohydrate-active enzyme annotation. Nucleic Acids Res..

[45] Rawlings ND, Barrett AJ, Bateman A (2010). MEROPS: the peptidase database. Nucleic Acids Res..

[46] Hunter S, Apweiler R, Attwood TK, Bairoch A, Bateman A, Binns D (2009). InterPro: the integrative protein signature database. Nucleic Acids Res.

[47] Roy A, Kucukural A, Zhang Y (2010). I-TASSER: a unified platform for automated protein structure and function prediction. Nat Protoc..

[48] Barragán MJL, Carmona M, Zamarro MT, Thiele B, Boll M, Fuchs G (2004). The bzd gene cluster, coding for anaerobic benzoate catabolism, in Azoarcus sp. strain CIB. J Bacteriol.

[49] Haft DH, Selengut JD, White O (2003). The TIGRFAMs database of protein families. Nucleic Acids Res.

[50] Finn RD, Bateman A, Clements J, Coggill P, Eberhardt RY, Eddy SR (2014). Pfam: the protein families database. Nucleic Acids Res..

[51] Stolz JF, Basu P (2002). Evolution of nitrate reductase: molecular and structural variations on a common function. Chembiochem..

[52] Søndergaard D, Pedersen CN, Greening C (2016). HydDB: a web tool for hydrogenase classification and analysis. Sci Rep..

[53] Kanehisa M, Goto S (2000). KEGG: kyoto encyclopedia of genes and genomes. Nucleic Acids Res..

[54] Marreiros BC, Batista AP, Duarte AM, Pereira MM (2013). A missing link between complex I and group 4 membrane-bound [NiFe] hydrogenases. Biochim Biophys Acta Bioenerg..

[55] Vignais PM, Billoud B (2007). Occurrence, classification, and biological function of hydrogenases: an overview. Chem Rev..

[56] Wolf YI, Makarova KS, Yutin N, Koonin EV (2012). Updated clusters of orthologous genes for Archaea: a complex ancestor of the Archaea and the byways of horizontal gene transfer. Biol Direct..

[57] Lesniewski RA, Jain S, Anantharaman K, Schloss PD, Dick GJ (2012). The metatranscriptome of a deep-sea hydrothermal plume is dominated by water column methanotrophs and lithotrophs. ISME J.

[58] Grabherr MG, Haas BJ, Yassour M, Levin JZ, Thompson DA, Amit I (2011). Full-length transcriptome assembly from RNA-Seq data without a reference genome. Nat Biotechnol.

[59] Ye J, McGinnis S, Madden TL (2006). BLAST: improvements for better sequence analysis. Nucleic Acids Res.

[60] Fu L, Niu B, Zhu Z, Wu S, Li W (2012). CD-HIT: accelerated for clustering the next-generation sequencing data. Bioinformatics..

[61] Payne KA, Quezada CP, Fisher K, Dunstan MS, Collins FA, Sjuts H (2015). Reductive dehalogenase structure suggests a mechanism for B12-dependent dehalogenation. Nature..

[62] Spang A, Stairs CW, Dombrowski N, Eme L, Lombard J, Caceres EF (2019). Proposal of the reverse flow model for the origin of the eukaryotic cell based on comparative analyses of Asgard archaeal metabolism. Nat Microbiol.

[63] Buchfink B, Xie C, Huson DH (2015). Fast and sensitive protein alignment using DIAMOND. Nat Methods..

[64] Li H, Durbin R (2009). Fast and accurate short read alignment with Burrows–Wheeler transform. Bioinformatics..

[65] Yarza P, Yilmaz P, Pruesse E, Glöckner FO, Ludwig W, Schleifer K-H (2014). Uniting the classification of cultured and uncultured bacteria and archaea using 16S rRNA gene sequences. Nat Rev Microbiol.

[66] Luo C, Rodriguez RL, Konstantinidis KT (2014). MyTaxa: an advanced taxonomic classifier for genomic and metagenomic sequences. Nucleic Acids Res..

[67] Harms U, Thauer RK (1996). Methylcobalamin: coenzyme M methyltransferase isoenzymes MtaA and MtbA from Methanosarcina barkeri: cloning, sequencing and differential transcription of the encoding genes, and functional overexpression of the mtaA gene in Escherichia coli. Eur J Biochem..

[68] Sauer K, Harms U, Thauer RK (1997). Methanol: coenzyme M methyltransferase from Methanosarcina barkeri: purification, properties and encoding genes of the corrinoid protein MT1. Eur J Biochem..

[69] Vanwonterghem I, Evans PN, Parks DH, Jensen PD, Woodcroft BJ, Hugenholtz P (2016). Methylotrophic methanogenesis discovered in the archaeal phylum Verstraetearchaeota. Nat Microbiol..

[70] Sofia HJ, Chen G, Hetzler BG, Reyes-Spindola JF, Miller NE (2001). Radical SAM, a novel protein superfamily linking unresolved steps in familiar biosynthetic pathways with radical mechanisms: functional characterization using new analysis and information visualization methods. Nucleic Acids Res..

[71] Callaghan A, Morris B, Pereira I, McInerney M, Austin RN, Groves JT (2012). The genome sequence of Desulfatibacillum alkenivorans AK-01: a blueprint for anaerobic alkane oxidation. Environ Microbiol..

[72] Herrmann G, Jayamani E, Mai G, Buckel W (2008). Energy conservation via electron-transferring flavoprotein in anaerobic bacteria. J Bacteriol..

[73] Weghoff MC, Bertsch J, Müller V (2015). A novel mode of lactate metabolism in strictly anaerobic bacteria. Environ Microbiol.

[74] Harwood CS, Burchhardt G, Herrmann H, Fuchs G (1998). Anaerobic metabolism of aromatic compounds via the benzoyl-CoA pathway. FEMS Microbiol Rev..

[75] Egland PG, Harwood CS (1999). BadR, a new MarR family member, regulates anaerobic benzoate degradation by Rhodopseudomonas palustris in concert with AadR, an Fnr family member. J Bacteriol..

[76] Ebenau-Jehle C, Boll M, Fuchs G (2003). 2-Oxoglutarate: NADP+ oxidoreductase in Azoarcus evansii: properties and function in electron transfer reactions in aromatic ring reduction. J Bacteriol..

[77] Peters JW, Schut GJ, Boyd ES, Mulder DW, Shepard EM, Broderick JB (2015). [FeFe]-and [NiFe]-hydrogenase diversity, mechanism, and maturation. Biochim Biophys Acta, Mol Cell Res..

[78] Bäumer S, Ide T, Jacobi C, Johann A, Gottschalk G, Deppenmeier U (2000). The F420H2 dehydrogenase from methanosarcina mazei is a redox-driven proton pump closely related to NADH dehydrogenases. J Biol Chem..

[79] Biegel E, Müller V (2010). Bacterial Na+-translocating ferredoxin: NAD+ oxidoreductase. Proc Natl Acad Sci USA..

[80] Buan NR, Metcalf WW (2010). Methanogenesis by Methanosarcina acetivorans involves two structurally and functionally distinct classes of heterodisulfide reductase. Mol Microbiol..

[81] Mand TD, Metcalf WW (2019). Energy conservation and hydrogenase function in methanogenic archaea, in particular the genus methanosarcina. Microbiol Mol Biol Rev..

[82] Heim S, Künkel A, Thauer RK, Hedderich R (1998). Thiol: fumarate reductase (Tfr) from Methanobacterium thermoautotrophicum: identification of the catalytic sites for fumarate reduction and thiol oxidation. Eur J Biochem.

[83] Mai X, Adams M (1994). Indolepyruvate ferredoxin oxidoreductase from the hyperthermophilic archaeon Pyrococcus furiosus. A new enzyme involved in peptide fermentation. J Biol Chem..

[84] Ma K, Hutchins A, Sung S-JS, Adams MW (1997). Pyruvate ferredoxin oxidoreductase from the hyperthermophilic archaeon, Pyrococcus furiosus, functions as a CoA-dependent pyruvate decarboxylase. Proc Natl Acad Sci USA..

[85] Ozawa Y, Siddiqui MA, Takahashi Y, Urushiyama A, Ohmori D, Yamakura F (2012). Indolepyruvate ferredoxin oxidoreductase: an oxygen-sensitive iron–sulfur enzyme from the hyperthermophilic archaeon Thermococcus profundus. J Biosci Bioeng..

[86] Moreno-Vivián C, Cabello P, Martínez-Luque M, Blasco R, Castillo F (1999). Prokaryotic nitrate reduction: molecular properties and functional distinction among bacterial nitrate reductases. J Bacteriol.

[87] McCready R, Gould W, Cook F (1983). Respiratory nitrate reduction by Desulfovibrio sp. Arch Microbiol..

[88] Lopez-Cortes A, Fardeau M-L, Fauque G, Joulian C, Ollivier B (2006). Reclassification of the sulfate-and nitrate-reducing bacterium Desulfovibrio vulgaris subsp. oxamicus as Desulfovibrio oxamicus sp. nov., comb. nov. Int J Syst Evol Microbiol..

[89] Leuthner B, Leutwein C, Schulz H, Hörth P, Haehnel W, Schiltz E (1998). Biochemical and genetic characterization of benzylsuccinate synthase from Thauera aromatica: a new glycyl radical enzyme catalysing the first step in anaerobic toluene metabolism. Mol Microbiol..

[90] Yang Y, Lin Y, Wang J, Wu Y, Zhang R, Cheng M (2018). Sensor-regulator and RNAi based bifunctional dynamic control network for engineered microbial synthesis. Nat Commun..

[91] Wegener G, Krukenberg V, Riedel D, Tegetmeyer HE, Boetius A (2015). Intercellular wiring enables electron transfer between methanotrophic archaea and bacteria. Nature..

[92] Srikantan S, Deng Y, Cheng Z-M, Luo A, Qin Y, Gao Q (2019). The tumor suppressor TMEM127 regulates insulin sensitivity in a tissue-specific manner. Nat Commun..

