# Supplementary material for: Newly discovered Asgard archaea Hermodarchaeota potentially degrade alkanes and aromatics via alkyl/benzyl-succinate synthase and benzoyl-CoA pathway
Source: ISME J. 2021 Jan 15;15(6):1826–43. doi: 10.1038/s41396-020-00890-x (PMC8163825; doi:10.1038/s41396-020-00890-x)
Supplement: Supplementary file 1 — Supplementary Tables and Figures [file 41396_2020_890_MOESM1_ESM.doc]

**Supplementary Information for**

**Newly discovered Asgard archaea Hermodarchaeota** **potentially** **degrade alkanes and aromatics via alkyl/benzyl-succinate synthase and benzoyl-CoA pathway**

Jia-Wei Zhang^1, 2^, Hong-Po Dong^1^*, Li-Jun Hou^1^*, Yang Liu^3^, Ya-Fei Ou^1^, Yan-Ling Zheng^4^, Ping Han^4^, Xia Liang^1^, Guo-Yu Yin^4^, Dian-Ming Wu^4^, Min Liu^4^, Meng Li^3^

^1^State Key Laboratory of Estuarine and Coastal Research, East China Normal University, Shanghai 200062, China; ^2^School of Ocean and Meteorology, Guangdong Ocean University, Zhanjiang 524088, China; ^3^Shenzhen Key Laboratory of Marine Microbiome Engineering, Institute for Advanced Study, Shenzhen University, Shenzhen 518060, China; ^4^Key Laboratory of Geographic Information Science, Ministry of Education, East China Normal University, Shanghai 200061, China

**Table of contents**

**A. Supplementary Methods 2-3**

1. Phylogenomic tree phylogeny

2. Sulfite reductase analyses

3. Sequence phylogeny of ribulose-1,5-bisphosphate carboxylase/oxygenase

4. Identification and phylogeny of Hermodarchaeota alkyl/benzyl-succinate synthase homologues

**B. Supplementary Discussion 3-7**

1. Carbon metabolism

2. System of energy conserving electron transport

3. Environmental distribution

**C. Supplementary Tables and Figures 8-32**

**Suppl. Tables 1-3, 5, 7, and Tables 10-12 8-17**

**Legends for Suppl. Tables 4, 6, 8-9, and 13 (provided in separate excel files) 18**

**Suppl. Figures 1-12 19-30**

**References 31-33**

**Supplementary Methods**

**1. Phylogenomic tree phylogeny**

The genome tree was also constructed using a concatenated set of 122 archaeal-specific marker genes in the Genome Taxonomy Database (GTDB, <https://gtdb.ecogenomic.org/>). The orthologs of these marker genes in the Hermodarchaeota and the reference genomes were identified using GTDB-Tk tool (v1.3.0, <https://github.com/Ecogenomics/GTDBTk>) based on hidden Markov models. Maximum-likelihood trees were constructed with IQ-TREE [1] using the following command: “-m TEST (LG+F+I+G4), -bb 1000”.

**2. Sulfite reductase analyses**

Identification of dissimilatory sulfite reductase (Dsr) and anaerobic sulfite reductase (Asr) genes, which are key enzymes for dissimilatory sulfate reduction, was performed by searching all predicted genes in Hermodarchaeota bins against *dsrA, dsrB, dsrD, asrA, asrB*, and *asrC* from TIGRfam [2] and Pfam [3] using hmmscan [4] (v.3.2.1). Motifs and conserved residues for DsrA, DsrB, DsrD, and AsrC proteins were analyzed by aligning the identified genes with known reference proteins [5]. Dozens of protein sequences were found to harbor Dsr or Asr domains. However, these sequences lacked strictly conserved binding sites for the siroheme-[4Fe4S] found in canonical DsrA, DsrB, and AsrC [5].

**3. Sequence phylogeny of ribulose-1,5-bisphosphate carboxylase/oxygenase**

Representative sequences of different forms of ribulose-1,5-bisphosphate carboxylase/oxygenase (Rubisco) reported previously by Tabita et al. [6], along with Rubisco sequences from Hermodarchaeota and other Asgard lineages, were aligned using MAFFT-L-INS-I [7] and trimmed using BMGE [8] (BLOSUM30 option). A maximum-likelihood phylogenic tree was inferred using IQtree [1] (v.1.6.12) under the LG+I+G4 model. Support values were calculated using 1,000 ultrafast bootstraps and SH-like approximate likelihood ratio test.

**4. Identification and phylogeny of Hermodarchaeota and bacterial alkyl/benzyl-succinate synthase** **homologues**

Metagenomic sequences from the six mangrove sediments collected from Techeng Island were *de novo* assembled individually using MEGAHIT [9] with the following parameters: --k-min 27--k-max 127--k-step 10. Protein coding sequences in the resulting contigs were predicted using Prodigal [10] (v.2.6.3) using prodigal -a pep.fas -c cds.fa -i contigs.fa -p meta. Alkyl/benzyl-succinate synthase (Ass/Bss) sequences were identified by searching all predicted genes against Hermodarchaeota and bacterial Ass/Bss sequences using Blastp [11] with an E-value < 1e-5. From the BLAST results, hits with a bit score ≥ 400 and length ≥ 650 amino acids were sorted and further filtered using CD-HIT [12] with a cut-off of 90% amino acid sequence identity. The motifs of Ass/Bss were analyzed following a previous method [13]. As a result, 394 sequences with archaeal “GC” motif and 34 sequences with bacterial “AC/LC” motif were obtained from the six metagenomic samples.

**B. Supplementary Discussion**

**1. Carbon metabolism**

Similar to other Asgard and Euryarchaeota archaea [14, 15], Hermodarchaeota possesses a modified Embden-Meyerhof pathway **(****Supplementary Table 9)**. It lacked canonical hexokinase catalyzing phosphorylation of glucose, but contained multiple sugar and carbohydrate kinases harboring the domain of the pfkB family carbohydrate kinase (PF00294), which have been found in other Asgard archaea. It is possible that these enzymes represent a new family of kinases capable of phosphorylating hexoses. We did not identify bifunctional ADP-dependent phosphofructokinase/glucokinases, which use ADP as the phosphoryl donor and have previously been identified in Heimdallarchaeota and several archaea of the Euryarchaeota [14, 15]. In addition, genes encoding a canonical glucose-6-phosphate isomerase were not detected in Hermodarchaeota; however, multiple genes for sugar phosphate isomerase/epimerase were present and may perform the same function **(Supplementary Table 9)**. Like several Asgard archaeal lineages, Hermodarchaeota contained genes encoding bifunctional archaeal fructose-1 6-bisphosphate aldolase (FBP)/phosphatase, with both FBP aldolase and FBP phosphatase activity [16], and canonical fructose-1, 6-bisphosphate phosphatase, suggesting alternative options in the gluconeogenesis reaction.

Members of Hermodarchaeota contained an incomplete tricarboxylic acid (TCA) cycle **(Fig. 2),** similar to Odinarchaeum and Lokiarchaeum [15]. All of the Hermodarchaeota genomes lacked genes for citrate synthase and citrate lyase while genes for aconitate hydratase or isocitrate dehydrogenase were only detected in one bin **(Fig. 2**, **Supplementary Table 9)**. This suggests that Hermodarchaeota is unable to oxidize acetyl-CoA derived from organic compounds into CO_2_ by the oxidative TCA cycle or to fix CO_2_ by the reductive TCA cycle. However, the incomplete TCA cycle may be able to mediate breakdown and synthesis of amino acids **(Fig. 2).**

Genes encoding all enzymes for the Wood–Ljungdahl pathway (WLP) were detected in each bin of Hermodarchaeota **(Fig. 2)**, similar to Thorarchaeota and Lokiarchaeum. The WLP consists of methyl and carbonyl branches [17]. In the carbonyl branch, a bifunctional and oxygen-sensitive enzyme complex, carbon monoxide dehydrogenase/acetyl-CoA synthase, consisting of five subunits, was identified in each genome of Hermodarchaeota **(Supplementary Table 9)**. The complex can catalyze both the reaction from CO, CoA, and methyl derived from the methyl branch to synthesize acetyl-CoA, and the reaction from acetyl-CoA to produce CO_2_ and methyl-H_4_MPT [18]. In methanogens, the methyl-H_4_MPT: coenzyme M methyltransferase (MTR) can transfer a methyl from H_4_MPT to coenzyme M (CoM) to form methyl-CoM coupling with translocation of Na^+^ across the membrane [19]. Subsequently, the methyl-CoM produced is converted to CH_4_ by methyl-coenzyme M reductase complex (MCR). The MTR complex consists of eight subunits [20]; however, only subunit H was found in Hermodarchaeota **(Fig. 2).** In addition, Hermodarchaeota lacked genes encoding all subunits of MCR. The feature is similar to all of the Asgard archaeal lineages except for Helarchaeota. This suggests that Hermodarchaeota is unable to perform methanogenesis. It is proposed that the presence of the archaeal WLP in the absence of MCR and MTR complexes might be the remnant of a previous combination with methanogenesis [19].

**2. System of energy conserving electron transport**

Genes encoding 11 subunits of NADH-quinone oxidoreductase were identified in Hermodarchaeota including *nuoA, B, C, D, H, I, J, K, L, M,* and *N* (**Supplementary Table 9)**, each subunit had multiple copies in one bin*.* However, in other Asgard archaea, only Heimdallarchaeota and Thorarchaeota possess a multisubunit NADH-quinone oxidoreductase (3–5 subunits) [15]. This enzyme, also called complex I, is an energy conserving enzyme that transfers electrons from NADH to quinones, coupled with translocation of protons across the membrane. The complex I and group 4 hydrogenase showed a close relationship. A previous method was used to differentiate between them [21]. According to this method, the NADH-quinone oxidoreductase of Hermodarchaeota lacked subunits EFG while no CxxC motifs were found in its nuoD; therefore, it should belong to complex I-related enzymes, but not group 4 hydrogenases. The presence of complex I suggests that Hermodarchaeota has potential to use NADH derived from the Embden-Meyerhof-Parnas pathway to produce energy.

Seven subunits of F_420_H_2_ dehydrogenase (Fpo DHLKMNJ) were found in the genomes of Hermodarchaeota (**Supplementary Table 9).** In contrast, multi-subunit F_420_H_2_ dehydrogenase has not been found in other Asgard archaeal lineages [15]. In methanogenic archaeon, F_420_H_2_ dehydrogenase is a membrane-bound energy conserving enzyme that is coupled to proton translocation across the membrane by catalyzing F_420_H_2_-dependent reduction of methanophenazine [22]. It is assumed that F_420_H_2_ dehydrogenase may be responsible for oxidation of F_420_H_2_ produced in oxidative WLP **(Fig. 2).**

Similar to other Asgard archaea, several genes encoding the large subunit of group 3 and group 4 [NiFe]-hydrogenases were identified in each genome of Hermodarchaeota via HydDB and phylogenetic analyses (**Supplementary Fig. 7)**. The N-terminal and C-terminal region of these hydrogenase sequences harbor a conserved CxxC motif (**Supplementary Fig. 7),** which can bind the [NiFe] centers for H_2_ oxidation [23]. In phylogenetic trees, most hydrogenase sequences from Hermodarchaeota can form a small cluster with those of other Asgard archaea. However, five group 3 hydrogenase sequences from Hermodarchaeota formed a novel cluster within group 3b (**Supplementary Fig. 7)**. Hermodarchaeota contained both group 3b and 3c hydrogenases. In the hyperthermophilic archaeon, *Pyrococcus furiosus*, group 3b hydrogenase possesses a NADP/FAD binding domain and can use H_2_ to reduce NADP for other biosynthetic metabolism [24] **(Fig. 2)**. In methanogens, group 3c hydrogenase is a methyl-viologen-reducing hydrogenase (Mvh) comprising three subunits (ADG). The enzyme is associated with heterodisulfide reductase (hdrABC) in the reduction of CoM-S-S-CoB [24] **(Fig. 2)**. The two group 3c hydrogenases of Hermodarchaeota were annotated as methyl-viologen-reducing hydrogenase alpha subunit (arCOG01549) in the arCOG database (**Supplementary Table 9).** The results suggest that Hermodarchaeota may be able to use H_2_ as electron donor. Phylogenetic analysis showed that most of group 4 hydrogenases of Hermodarchaeota belonged to group 4g (**Supplementary Fig. 7)**, which is a H_2_-evolving membrane-bound hydrogenase. In *P. furiosus,* a 14-subunit membrane bound hydrogenase (MBH) oxidizes reduced ferredoxin generated by sugar fermentation and releases H_2_ coupled to translocation of sodium ion across the cell membrane [25]. It has been found that many subunits for MBH are homologous to subunits of complex I and Mrp H^+^/Na^+^ antiporter [26]. As mentioned above, many subunits in complex I for each genome of Hermodarchaeota had multiple copies in which some possessed the domain of the Mrp antiporter (such as nuoLMN) (IPR001750). It is likely that some subunit copies in complex I may be a part of the MBH complex in Hermodarchaeota. Thus, members of Hermodarchaeota may be capable of using MBH to establish a Na^+^ gradient for ATP synthesis **(Fig. 2).**

Each genome of Hermodarchaeota contained genes for reductive dehalogenase. These enzymes were closely related to reductive dehalogenase from Bathyarchaeota archaeon and Verstraetearchaeota archaeon (E-value: 2.88E-151 to 6.56E-151; bit score: 439), and contained a domain of reductive dehalogenase (IPR028894). Reductive dehalogenases perform dehalogenation in organohalide respiring bacteria that are responsible for the decomposition of many organohalide pollutants, such as polychlorinated biphenyls and dioxins [27, 28]. Phylogenetic analyses using a previous dataset with functionally characterized reductive dehalogenases [29] revealed that reductive dehalogenases of Hermodarchaeota were grouped with those of Lokiarchaeota, Thorarchaeota, and Heimdalarchaeota, as well as of *Dehalospirillum multivorans* PceA (O68252) and *Desulfitobacterium* sp. PCE-1 (**Supplementary Fig. 11),** suggesting that these Asgard archaea may be able to utilize chlorinated ethenes/ethanes as electron acceptors.

**3. Environmental distribution**

Although gene fragments homologous to Hermodarchaeota alkyl/benzyl-succinate synthase (Ass/Bss) were detected in a variety of environments, they were mainly found in high abundance in metagenomes from deep sea petroleum seepages, marine hydrothermal vents, hot springs, mangrove swamps, lake sediments, and formation water from coal seams, while only detected in low abundance in metagenomes from deep-sea water, Amazon forest soil, and freshwater wetland sediments (**Supplementary Table 6; Fig. 6b).** This appears to be consistent with the content of a variety of alkanes or aromatic hydrocarbons contained in these environments. The presence of abundant Ass/Bss genes in many mangrove wetlands may be due to release of large amount of lignin-derived phenols and n-alkanes from mangrove tissues as well as aromatic compounds produced by lignin degradation [30]. The Hermodarchaeota 16S rRNA gene was identified in most of these environments, implicating ubiquity of members of Hermodarchaeota in nature. In certain habitats, the high abundance of Ass/Bss gene fragments corresponded to a high percentage of the Hermodarchaeota 16S rRNA gene (**Supplementary Table 6; Fig. 6b)**, suggesting a substantial contribution of Hermodarchaeota to anaerobic degradation of alkanes or aromatic hydrocarbons.

**Supplementary Table 1.** Sediment characteristics of sampling sites.

| Samples | Location | Depth  (m) | pH | Eh  (mV) | DO  (mg L^-1^) | NH_4_^+^  (μg g^-1^) | NO_3_^-^  (μg g^-1^) | NO_2_^-^  (μg g^-1^) | Benzene  (μg kg^-1^) | Toluene  (μg kg^-1^) |
| --- | --- | --- | --- | --- | --- | --- | --- | --- | --- | --- |
| h02s | H02, 110°26′45″, 21°10′27″ | 0.15-0.20 | 7.4 | -311.02 | ND | 10.39 | 0.59 | 0.14 | ND | 3.05 |
| h02m |  | 0.40-0.45 | 7.1 | -230.73 | ND | 9.94 | 0.64 | 0.08 | ND | 3.28 |
| h02b |  | 0.95-1.0 | 6.7 | -197.25 | ND | 9.85 | 0.58 | 0.08 | 5.73 | 15.14 |
| h03s | H03, 110°26′19.67″,  21°9′10.80″ | 0.15-0.20 | 7.4 | -254.35 | ND | 10.67 | 0.57 | 0.06 | 1.01 | 23.30 |
| h03m |  | 0.40-0.45 | 6.6 | -253.92 | ND | 10.36 | 0.56 | 0.04 | 5.28 | 24.9 |
| h03b |  | 0.95-1.0 | 6.3 | -167.44 | ND | 9.76 | 0.72 | 0.07 | 1.80 | 25.7 |

^*^Sediment pH, redox potential (Eh), and dissolved oxygen (DO) were in triplicate measured using a micromanipulator meter **system (Unisense,** Denmark) with a needle pH sensor, a needle Eh senor and a needle oxygen sensor, respectively. Sediment ammonium (NH_4_^+^), nitrate (NO_3_^–^) and nitrite (NO_2_^–^) were extracted by 2 mol L^-1^ KCl, and their concentrations were determined via flow injection analysis (Skalar Analytical SAN++, Netherlands). Aromatic compounds were determined using gas chromatography-mass spectrometry. Ethylbenzene, p-Xylene, m-Xylene, o-Xylene, and phenylethylene were not detected. ND represents the values below the detection limit.

**Supplementary Table 2.** Statistics of Asgard archaeal bins recovered from mangrove sediment samples

| **Bin ID** | **Assembly tool used** | **Completeness (%)** | **Contamination (%)** | **Strain heterogeneity (%)** | **NO. of gene** | **GC content (%)** | **NO. of Scaffold**  **/contigs** | **Genome Size**  **(Mbp)** | **Largest scaffold/contig**  **(bp)** | **Taxonomy** |
| --- | --- | --- | --- | --- | --- | --- | --- | --- | --- | --- |
| h02s_68 | Megahit | 86.22 | 0.47 | 0 | 4833 | 43.91 | 903 | 5.1 | 46141 | Hermodarchaeota |
| h02s_80 | Megahit | 92.67 | 0 | 0 | 3636 | 44.54 | 267 | 3.76 | 99342 | Hermodarchaeota |
| h02s_124 | Megahit | 73.33 | 0 | 0 | 2418 | 48.97 | 338 | 2.45 | 48037 | Hermodarchaeota |
| h02m_52 | Megahit | 77.46 | 0 | 0 | 2582 | 44.52 | 357 | 2.68 | 68536 | Hermodarchaeota |
| h02m_117 | Megahit | 74.69 | 0 | 0 | 1785 | 44.71 | 269 | 1.86 | 36381 | Hermodarchaeota |
| h02m_131 | Megahit | 89.5 | 1.87 | 0 | 3895 | 43.21 | 863 | 4.22 | 54136 | Hermodarchaeota |
| h03m_104 | Megahit | 78.04 | 0 | 0 | 2561 | 43.05 | 469 | 2.66 | 32924 | Hermodarchaeota |
| h02s_26 | metaSPAdes | 76.42 | 1.94 | 0 | 2508 | 48.74 | 677 | 2.53 | 28150 | Hermodarchaeota |
| h02s_84 | Megahit | 73.39 | 2.96 | 0 | 1722 | 38.15 | 442 | 1.78 | 11729 | Heimdallarchaeota |
| h03b_10 | Megahit | 79.16 | 2.49 | 0 | 1666 | 37.69 | 696 | 1.73 | 10281 | Heimdallarchaeota |
| h02m_144 | Megahit | 77.93 | 0.93 | 100 | 3393 | 29.81 | 640 | 3.55 | 56443 | Lokiarchaeota |
| h02m_142 | Megahit | 90.63 | 0 | 0 | 2749 | 42.7 | 306 | 2.66 | 69294 | Odinarchaeota |
| h02s_33 | Megahit | 60.85 | 0 | 0 | 2289 | 44.29 | 564 | 2.36 | 13267 | Thorarchaeota |

**Supplementary Table 3.** 16S rRNA genes of Hermodarchaeota.

| **ID** | **Samples** | **Assembly tool** | **Scaffold/Contig length** | **Position of 16S rRNA gene** | **Length of 16S rRNA gene** | **Bin of Hermodarchaeota** |
| --- | --- | --- | --- | --- | --- | --- |
| h02s.26_NODE_7530 | h02s | metaSPAdes | 3416 | 1-1066 | 1066 | h02s_26 |
| h02m_131_k127_628877 | h02m | Megahit | 2810 | 2305-2810 | 506 | h02m_131 |
| h02s_matam_3940 | h02s | MATAM | - | - | 1450 | - |
| h02s_matam_8111 | h02s | MATAM | - | - | 879 | - |

**Supplementary Table 5.** List of 56 ribosomal proteins used for inference of phylogenomic trees in this study.

| **arCOG** | **COG** | **Description** |
| --- | --- | --- |
| arCOG04086 | COG1841 | Ribosomal protein L30/L7E |
| arCOG00779 | COG0200 | Ribosomal protein L15 |
| arCOG04087 | COG0098 | Ribosomal protein S5 |
| arCOG04154 | COG2007 | Ribosomal protein S8E |
| arCOG04088 | COG0256 | Ribosomal protein L18 |
| arCOG04372 | COG0080 | Ribosomal protein L11 |
| arCOG01751 | COG1358 | Ribosomal protein L7Ae or related RNA K-turn-binding protein |
| arCOG04090 | COG0097 | Ribosomal protein L6P/L9E |
| arCOG04242 | COG0102 | Ribosomal protein L13 |
| arCOG04245 | COG0052 | Ribosomal protein S2 |
| arCOG04243 | COG0103 | Ribosomal protein S9 |
| arCOG04089 | COG2147 | Ribosomal protein L19E |
| arCOG04091 | COG0096 | Ribosomal protein S8 |
| arCOG04093 | COG1471 | Ribosomal protein S4E |
| arCOG04094 | COG0198 | Ribosomal protein L24 |
| arCOG04095 | COG0093 | Ribosomal protein L14 |
| arCOG04289 | COG0081 | Ribosomal protein L1 |
| arCOG00780 | COG1727 | Ribosomal protein L18E |
| arCOG00781 | COG1717 | Ribosomal protein L32E |
| arCOG04067 | COG0090 | Ribosomal protein L2 |
| arCOG04070 | COG0087 | Ribosomal protein L3 |
| arCOG04071 | COG0088 | Ribosomal protein L4 |
| arCOG04092 | COG0094 | Ribosomal protein L5 |
| arCOG04096 | COG0186 | Ribosomal protein S17 |
| arCOG04288 | COG0244 | Ribosomal protein L10 |
| arCOG04072 | COG0089 | Ribosomal protein L23 |
| arCOG04097 | COG0092 | Ribosomal protein S3 |
| arCOG04098 | COG0091 | Ribosomal protein L22 |
| arCOG04239 | COG0522 | Ribosomal protein S4 or related protein |
| arCOG04314 | COG2053 | Ribosomal protein S28E/S33 |
| arCOG04182 | COG2004 | Ribosomal protein S24E |
| arCOG01946 | COG2125 | Ribosomal protein S6E (S10) |
| arCOG04129 | COG2139 | Ribosomal protein L21E |
| arCOG04186 | COG1890 | Ribosomal protein S3AE |
| arCOG04255 | COG0048 | Ribosomal protein S12 |
| arCOG00785 | COG0255 | Ribosomal protein L29 |
| arCOG01752 | COG1841 | Ribosomal protein L30/L7E |
| arCOG04208 | COG1997 | Ribosomal protein L37AE/L43A |
| arCOG01758 | COG0051 | Ribosomal protein S10 |
| arCOG04113 | COG0197 | Ribosomal protein L16/L10AE |
| arCOG04240 | COG0100 | Ribosomal protein S11 |
| arCOG04254 | COG0049 | Ribosomal protein S7 |
| arCOG04287 | COG2058 | Ribosomal protein L12E/L44/L45/RPP1/RPP2 |
| arCOG04099 | COG0185 | Ribosomal protein S19 |
| arCOG04185 | COG0184 | Ribosomal protein S15P/S13E |
| arCOG01722 | COG0099 | Ribosomal protein S13 |
| arCOG04209 | COG1632 | Ribosomal protein L15E |
| arCOG04473 | COG2097 | Ribosomal protein L31E |
| arCOG01885 | COG1383 | Ribosomal protein S17E |
| arCOG01344 | COG2238 | Ribosomal protein S19E (S16A) |
| arCOG04108 | COG2051 | Ribosomal protein S27E |
| arCOG04109 | COG1631 | Ribosomal protein L44E |
| arCOG04167 | COG0093 | Ribosomal protein L14 |
| arCOG04183 | COG1998 | Ribosomal protein S27AE |
| arCOG01950 | COG2075 | Ribosomal protein L24E |
| arCOG04175 | COG2157 | Ribosomal protein L20A (L18A) |

**Supplementary Table 7.** Blastn output results retrieved by comparing Hermodarchaeota 16S rRNA gene sequence with those from sediment samples and published Asgard archaea.

| **Query id** | **Subject id** | **% identity** | **alignment length** | **mismatches** | **gap openings** | **q. start** | **q. end** | **s. start** | **s. end** | **evalue** | **bit score** |
| --- | --- | --- | --- | --- | --- | --- | --- | --- | --- | --- | --- |
| h02s_26_NODE_7530 | h02s_matam_3940 | 95.775 | 1065 | 43 | 2 | 3 | 1066 | 1 | 1064 | 0 | 1716 |
| h02s_26_NODE_7530 | h02s_matam_8111 | 95.108 | 879 | 43 | 0 | 69 | 947 | 1 | 879 | 0 | 1386 |
| h02s_26_NODE_7530 | Odinarchaeote-LCB_4_MDVT01000007.1 | 83.711 | 927 | 141 | 10 | 145 | 1066 | 186 | 1107 | 0 | 867 |
| h02s_26_NODE_7530 | Lokiarchaeota-archaeon-B53_G9SDNY01000025.1 | 79.835 | 848 | 152 | 18 | 227 | 1066 | 219 | 1055 | 1.46E-174 | 601 |
| h02s_26_NODE_7530 | Lokiarchaeum-sp.-GC14_75_JYIM01000321.1 | 79.762 | 756 | 129 | 22 | 321 | 1066 | 1 | 742 | 2.52E-152 | 527 |
| h02s_26_NODE_7530 | Thorarchaeota-archaeon-MP11T_1_PJET01000033.1 | 77.39 | 774 | 150 | 20 | 286 | 1046 | 281 | 1042 | 4.36E-125 | 436 |
| h02s_26_NODE_7530 | Thorarchaeota-archaeon-MP8T_1_PJER01000019.1 | 77.261 | 774 | 151 | 22 | 286 | 1046 | 281 | 1042 | 2.03E-123 | 431 |
| h02s_26_NODE_7530 | Helarchaeota_Ga0180301_100789461 | 81.683 | 404 | 64 | 10 | 480 | 878 | 1 | 399 | 2.74E-92 | 327 |
| h02s_26_NODE_7530 | Heimdalarchaeote-LC_3_MDVS01000157.1 | 72.931 | 713 | 162 | 28 | 369 | 1066 | 6 | 702 | 1.72E-59 | 219 |
| h02m_131_k127_628877 | Odinarchaeote-LCB_4_MDVT01000007.1 | 79.921 | 508 | 72 | 21 | 1 | 505 | 1063 | 1543 | 3.53E-98 | 346 |
| h02m_131_k127_628877 | Helarchaeota_Ga0180301_100789461 | 77.559 | 508 | 84 | 17 | 1 | 505 | 577 | 1057 | 3.63E-78 | 279 |
| h02m_131_k127_628877 | Lokiarchaeum-sp.-GC14_75_JYIM01000321.1 | 78.61 | 374 | 75 | 4 | 1 | 372 | 698 | 1068 | 4.77E-67 | 243 |
| h02m_131_k127_628877 | Lokiarchaeota-archaeon-B53_G9SDNY01000025.1 | 77.807 | 374 | 78 | 4 | 1 | 372 | 1011 | 1381 | 4.8E-62 | 226 |

**Supplementary Table 10.** Comparison of alkylsuccinate synthase (Ass)/benzylsuccinate synthase (Bss) of Hermodarchaeota with known Ass/Bss and pyruvate formate lyase (pfl) using Blastp.

| **Query id** | **Subject id** | **% identity** | **alignment length** | **mismatches** | **gap openings** | **q. start** | **q. end** | **s. start** | **s. end** | **evalue** | **bit score** |
| --- | --- | --- | --- | --- | --- | --- | --- | --- | --- | --- | --- |
| h02s_68 k137_1286063_3 | AssA1 from *D. alkenivorans* strain AK-01(ABH11460) | 33.38 | 605 | 380 | 12 | 52 | 642 | 235 | 830 | 1.18E-98 | 312 |
| h02s_68 k137_1286063_3 | BssA from *A. aromaticum* EbN1 (YP_158060) | 31.72 | 618 | 384 | 16 | 52 | 643 | 241 | 846 | 3.93E-87 | 281 |
| h02s_68 k137_1286063_3 | pfl from *E. coli* (NP_415423) | 25.08 | 650 | 414 | 20 | 10 | 646 | 169 | 758 | 3.94E-46 | 165 |
| h02s_68 k137_1286063_3 | pflD from *Archaeoglobus fulgidus* (AAB89800) | 38.15 | 637 | 383 | 8 | 144 | 771 | 7 | 641 | 8.74e-158 | 463 |
| h02m_131 k127_726590_1 | AssA1 from *D. alkenivorans* strain AK-01(ABH11460) | 29.22 | 705 | 447 | 14 | 128 | 794 | 139 | 829 | 7.03E-79 | 262 |
| h02m_131 k127_726590_1 | BssA from *A. aromaticum* EbN1 (YP_158060) | 30.65 | 620 | 373 | 18 | 209 | 794 | 248 | 844 | 1.03E-68 | 234 |
| h02m.131 k127_726590_1 | pfl from *E. coli* (NP_415423) | 27.29 | 590 | 354 | 21 | 229 | 794 | 216 | 754 | 2.21E-38 | 143 |
| h02m_131 k127_726590_1 | pflD from *Archaeoglobus fulgidus* (AAB89800) | 33.44 | 646 | 383 | 16 | 155 | 773 | 171 | 796 | 3.66e-107 | 336 |
| h02s_68 k137_157306_13 | AssA1 from *D. alkenivorans* strain AK-01(ABH11460) | 30.51 | 790 | 505 | 19 | 27 | 789 | 60 | 832 | 2.28E-96 | 310 |
| h02s_68 k137_157306_13 | BssA from *A. aromaticum* EbN1 (YP_158060) | 29.96 | 801 | 502 | 23 | 27 | 790 | 70 | 848 | 3.24E-90 | 294 |
| h02s_68 k137_157306_13 | pfl from *E. coli* (NP_415423) | 24.55 | 554 | 356 | 16 | 257 | 792 | 251 | 760 | 3.49E-29 | 114 |
| h02s_68 k137_157306_13 | pflD from *Archaeoglobus fulgidus* (AAB89800) | 33.80 | 787 | 498 | 17 | 4 | 773 | 8 | 788 | 8.06e-140 | 421 |
| h02m.117_k127_21078_1 | AssA1 from *D. alkenivorans* strain AK-01(ABH11460) | 31.313 | 693 | 438 | 16 | 54 | 719 | 151 | 832 | 9.71E-92 | 294 |
| h02m.117_k127_21078_1 | BssA from *A. aromaticum* EbN1 (YP_158060) | 29.062 | 757 | 480 | 22 | 1 | 720 | 112 | 848 | 2.30E-81 | 266 |
| h02m.117_k127_21078_1 | pfl from *E. coli* (NP_415423) | 24.231 | 553 | 359 | 15 | 187 | 722 | 251 | 760 | 2.25E-29 | 113 |
| h02m.117_k127_21078_1 | pflD from *Archaeoglobus fulgidus* (AAB89800) | 33.84 | 724 | 459 | 15 | 1 | 718 | 64 | 773 | 2.01E-131 | 398 |
| h02m.52_k127_1722569_10 | AssA1 from *D. alkenivorans* strain AK-01(ABH11460) | 31.512 | 787 | 501 | 17 | 23 | 785 | 60 | 832 | 1.47E-100 | 320 |
| h02m.52_k127_1722569_10 | BssA from *A. aromaticum* EbN1 (YP_158060) | 29.287 | 799 | 510 | 25 | 23 | 786 | 70 | 848 | 6.42E-90 | 291 |
| h02m.52_k127_1722569_10 | pfl from *E. coli* (NP_415423) | 23.547 | 688 | 433 | 20 | 124 | 788 | 143 | 760 | 4.03E-30 | 115 |
| h02m.52_k127_1722569_10 | pflD from *Archaeoglobus fulgidus* (AAB89800) | 34.008 | 791 | 497 | 18 | 1 | 784 | 1 | 773 | 2.61E-142 | 428 |

**Supplementary Table 11.** Comparison of alkylsuccinate synthase/benzylsuccinate synthase activating enzyme (Ass/Bss AE) of Hermodarchaeota with known Ass/Bss and pyruvate formate lyase (pfl) AE using Blastp.

| **Query id** | **Subject id** | **% identity** | **alignment length** | **mismatches** | **gap openings** | **q. start** | **q. end** | **s. start** | **s. end** | **evalue** | **bit score** |
| --- | --- | --- | --- | --- | --- | --- | --- | --- | --- | --- | --- |
| h02s.80 k137_2455425_12 | AssD2 from *D. alkenivorans* strain AK-01 (YP_002431363) | 39.16 | 286 | 160 | 3 | 1 | 276 | 19 | 300 | 1.30E-67 | 203 |
| h02s.80 k137_2455425_12 | AssD2' from *D. alkenivorans* strain AK-01 (YP_002429341) | 40.94 | 298 | 167 | 4 | 1 | 289 | 17 | 314 | 3.44E-86 | 250 |
| h02s.80 k137_2455425_12 | PflC from *Archaeoglobus fulgidus* (KUJ94427) | 40.48 | 252 | 146 | 2 | 1 | 252 | 20 | 267 | 3.56E-68 | 204 |
| h02s.80 k137_2455425_12 | BssD from *T. aromatica* K172 (CAA05050) | 38.46 | 260 | 152 | 4 | 1 | 253 | 18 | 276 | 3.47E-65 | 197 |
| h02s.80 k137_2455425_12 | pflD from *E.coli* (NP_415422) | 31.64 | 177 | 118 | 2 | 86 | 260 | 48 | 223 | 1.08E-31 | 107 |
| h02s.68 k137_3621160_2 | AssD2 from *D. alkenivorans* strain AK-01 (YP_002431363) | 42.91 | 282 | 151 | 3 | 23 | 294 | 6 | 287 | 9.42E-75 | 223 |
| h02s.68 k137_3621160_2 | AssD2' from *D. alkenivorans* strain AK-01 (YP_002429341) | 44.87 | 312 | 163 | 4 | 22 | 324 | 3 | 314 | 8.71E-98 | 281 |
| h02s.68 k137_3621160_2 | PflC from *Archaeoglobus fulgidus* (KUJ94427) | 41.26 | 269 | 154 | 2 | 19 | 287 | 3 | 267 | 1.54E-72 | 216 |
| h02s.68 k137_3621160_2 | BssD from *T. aromatica* K172 (CAA05050) | 37.59 | 274 | 165 | 2 | 20 | 287 | 2 | 275 | 1.31E-68 | 207 |
| h02s.68 k137_3621160_2 | pflD from *E.coli* (NP_415422) | 31.82 | 264 | 121 | 4 | 21 | 282 | 4 | 210 | 3.92E-40 | 131 |
| h02m.131 k127_726590_2 | AssD2 from *D. alkenivorans* strain AK-01 (YP_002431363) | 39.37 | 287 | 164 | 1 | 7 | 283 | 2 | 288 | 1.60E-73 | 218 |
| h02m.131 k127_726590_2 | AssD2' from *D. alkenivorans* strain AK-01 (YP_002429341) | 45.33 | 300 | 157 | 3 | 10 | 302 | 3 | 302 | 7.74E-100 | 285 |
| h02m.131 k127_726590_2 | PflC from *Archaeoglobus fulgidus* (KUJ94427) | 40.23 | 266 | 155 | 2 | 10 | 275 | 6 | 267 | 2.08E-72 | 215 |
| h02m.131 k127_726590_2 | BssD from *T. aromatica* K172 (CAA05050) | 38.38 | 271 | 161 | 3 | 12 | 276 | 6 | 276 | 9.81E-68 | 204 |
| h02m.131 k127_726590_2 | pflD from *E.coli* (NP_415422) | 30.42 | 263 | 124 | 5 | 10 | 270 | 5 | 210 | 3.86E-37 | 122 |
| h02m.52_k127_2232651_3 | AssD2 from *D. alkenivorans* strain AK-01 (YP_002431363) | 39.597 | 298 | 166 | 3 | 34 | 321 | 7 | 300 | 8.51E-71 | 213 |
| h02m.52_k127_2232651_3 | AssD2' from *D. alkenivorans* strain AK-01 (YP_002429341) | 41.534 | 313 | 174 | 4 | 31 | 334 | 2 | 314 | 2.79E-94 | 273 |
| h02m.52_k127_2232651_3 | PflC from *Archaeoglobus fulgidus* (KUJ94427) | 42.857 | 266 | 148 | 2 | 32 | 297 | 6 | 267 | 9.73E-76 | 225 |
| h02m.52_k127_2232651_3 | BssD from *T. aromatica* K172 (CAA05050) | 39.706 | 272 | 156 | 4 | 34 | 298 | 6 | 276 | 6.09E-68 | 206 |
| h02m.52_k127_2232651_3 | pflD from *E.coli* (NP_415422) | 28.571 | 273 | 136 | 6 | 32 | 302 | 5 | 220 | 7.63E-33 | 111 |
| h02s.26_NODE_881_7 | AssD2 from *D. alkenivorans* strain AK-01 (YP_002431363) | 38.614 | 303 | 172 | 2 | 45 | 337 | 2 | 300 | 7.16E-73 | 218 |
| h02s.26_NODE_881_7 | AssD2' from *D. alkenivorans* strain AK-01 (YP_002429341) | 46.333 | 300 | 154 | 4 | 48 | 340 | 3 | 302 | 7.32E-95 | 275 |
| h02s.26_NODE_881_7 | PflC from *Archaeoglobus fulgidus* (KUJ94427) | 42.322 | 267 | 150 | 2 | 48 | 314 | 6 | 268 | 1.56E-72 | 217 |
| h02s.26_NODE_881_7 | BssD from *T. aromatica* K172 (CAA05050) | 38.889 | 270 | 159 | 2 | 49 | 312 | 5 | 274 | 1.28E-68 | 208 |
| h02s.26_NODE_881_7 | pflD from *E.coli* (NP_415422) | 33.69 | 187 | 121 | 2 | 147 | 331 | 48 | 233 | 8.80E-32 | 109 |

**Supplementary Table 12** Genes encoding alkyl/benzyl-succinate synthase and benzoyl-CoA reductase similar to those of Hermodarchaeota that were identified from an assembled metatranscriptome (SRR11241197) generated from mangrove sediment using Blastx.

| **Query id** | **Query sequence length (bp)** | **Subject id** | **gene** | **Subject sequence length (aa)** | **identity%** | **alignment length (aa)** | **coverage** | **e-value** | **bit score** |
| --- | --- | --- | --- | --- | --- | --- | --- | --- | --- |
| TRINITY_DN106007_c1_g2_i3 | 1067 | h02s.68@k137_157306_13 | *Ass/Bss* | 799 | 60.423 | 331 | 93 | 5.81E-142 | 412 |
| TRINITY_DN106007_c1_g4_i1 | 877 | h02m.52@k127_1722569_10 | *Ass/Bss* | 795 | 61.168 | 291 | 99 | 1.79E-127 | 372 |
| TRINITY_DN384218_c0_g1_i1 | 593 | h02s_68_k137_3447571_2 | *BzdQ（bcrA）* | 296 | 60.606 | 198 | 99 | 1.92E-87 | 250 |
| TRINITY_DN1722429_c0_g1_i1 | 482 | h03m_104_k127_2992571_4 | *BzdN（bcrC）* | 374 | 62.658 | 158 | 98 | 1.04E-68 | 203 |

**Legends for Suppl. Tables 4, 6, 8-9, and 13.**

**Please note that these Suppl. Tables are provided in separate excel files.**

**Supplementary Table 4.** List of organisms used in genome trees.

**Supplementary Table 6.** Metagenomic samples from a variety of environments containing homologues of Hermodarchaeota alkyl/benzyl-succinate synthase (Ass/Bss) and/or 16S rRNA genes.

**Supplementary Table 8.** Distribution of eukaryotic signature proteins in Hermodarchaeota and other Asgard archaea.

**Supplementary Table 9.** The genes used for metabolic reconstruction in this study.

**Supplementary Table 13.** Features of homologs of Hermodarchaeota and bacterial alkyl/benzyl-succinate synthase (Ass/Bss) identified using Blasp in metagenomes generated from the six sediment samples from mangrove swamps in Techeng Island, China.


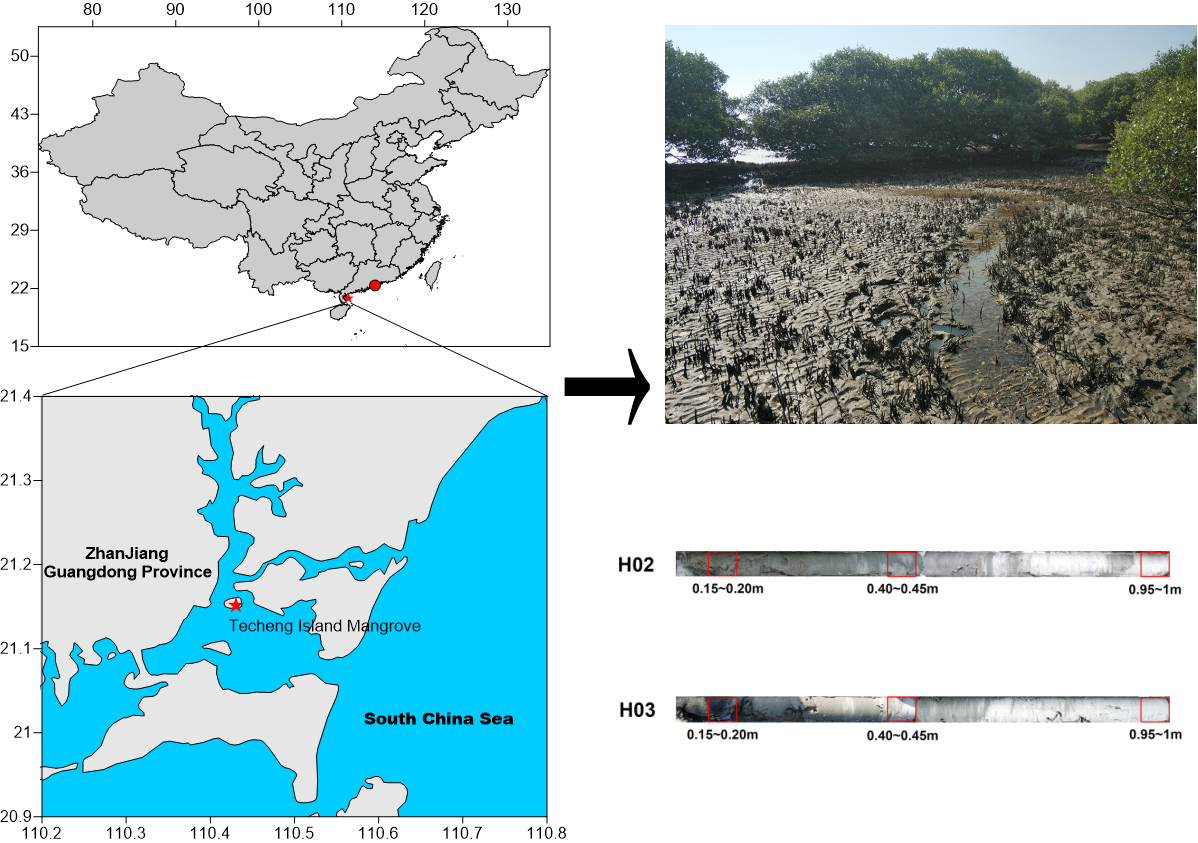


**Supplementary Fig. 1** Geographic locations of the sampling sites from mangrove swamps. The red stars represent the sites for metagenomic data in this study. The red dot represents the sites for metatranscriptomic data which are downloaded from NCBI database [31].


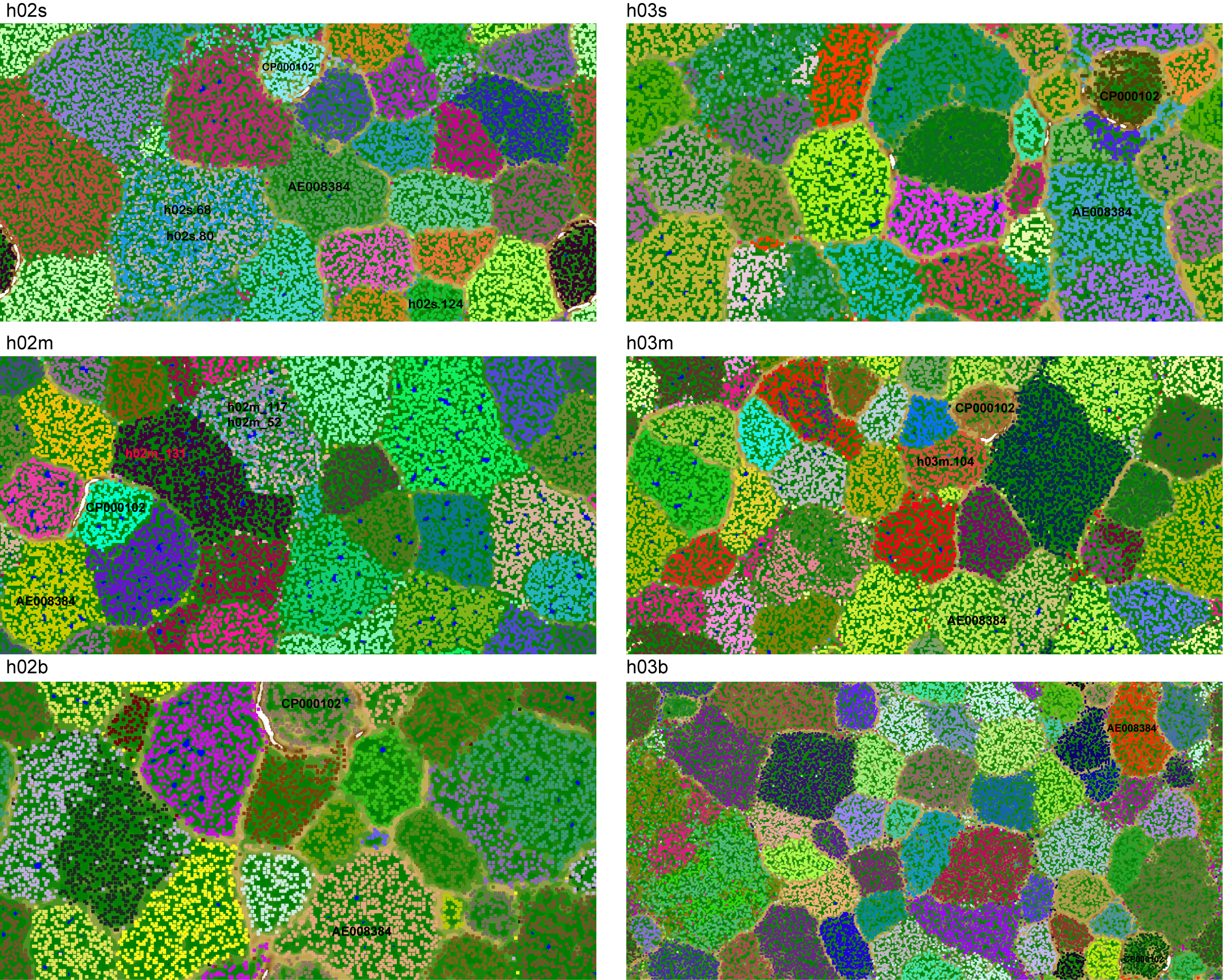


**Supplementary Fig. 2** Visualization of bins assembled from six mangrove sediment samples using Emergent Self-Organizing map (ESOM). Seven Hermodarchaeota bins were indicated, including h02s.80, h02m.131, h02s.68, h02s.124, h02m.52, h02m.117 and h03m_104. AE008384 and CP000102 represent genomes of *Methanosarchina mazei* strain Goe1 [32] and *Methanosphaera stadtmanae* [33]*,* respectively, and they were used as reference genomes.


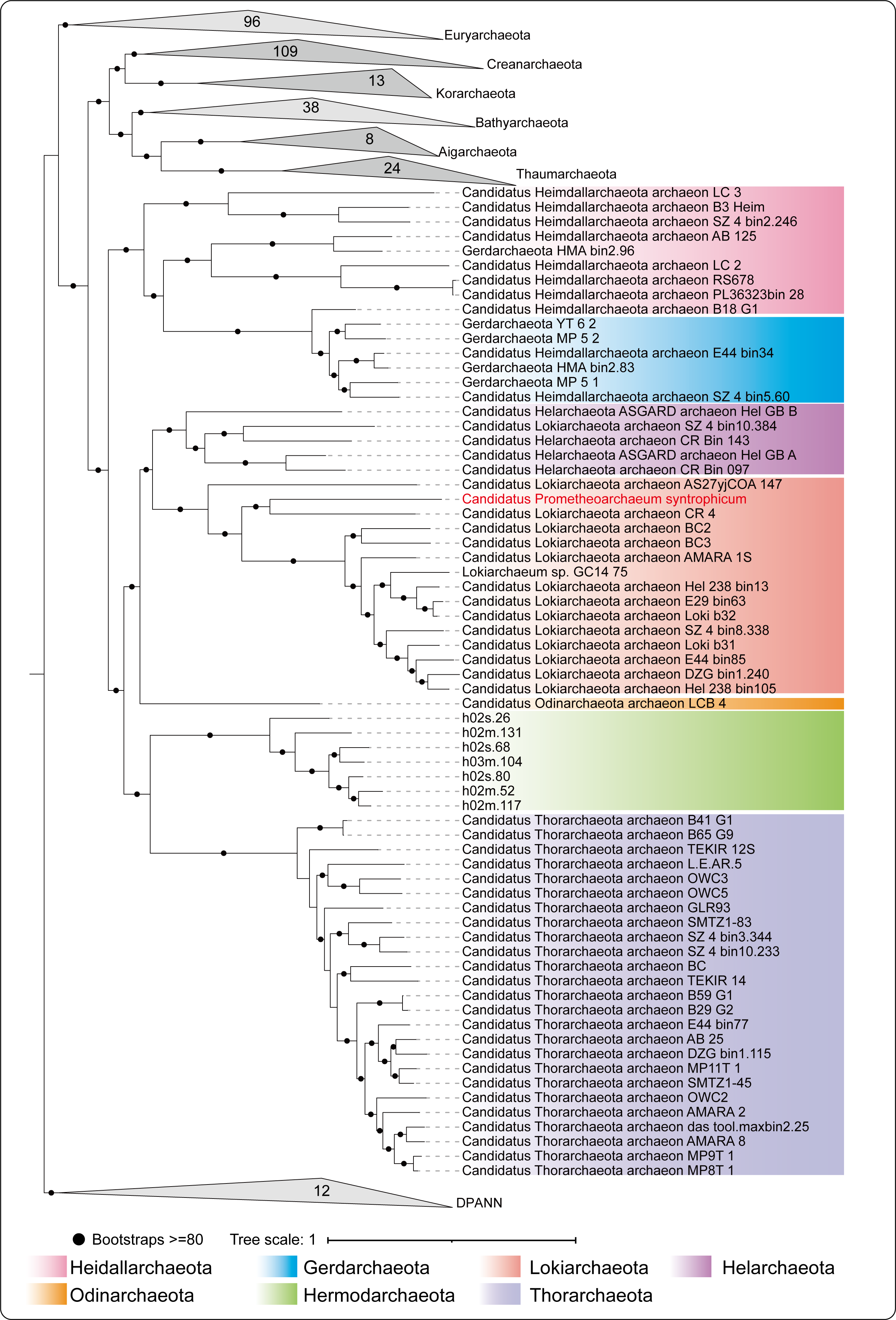


**Supplementary Fig. 3** Phylogenomic tree showing the placement of Hermodarchaeota relative to 361 archaeal genomes, using the DPANN superphylum as the outgroup. The tree was reconstructed using IQtree (LG+F+I+G4) with a concatenated set of 122 archaeal-specific marker proteins. Nodes with ultrafast bootstrap values ≥ 80 are indicated by black circles.

**
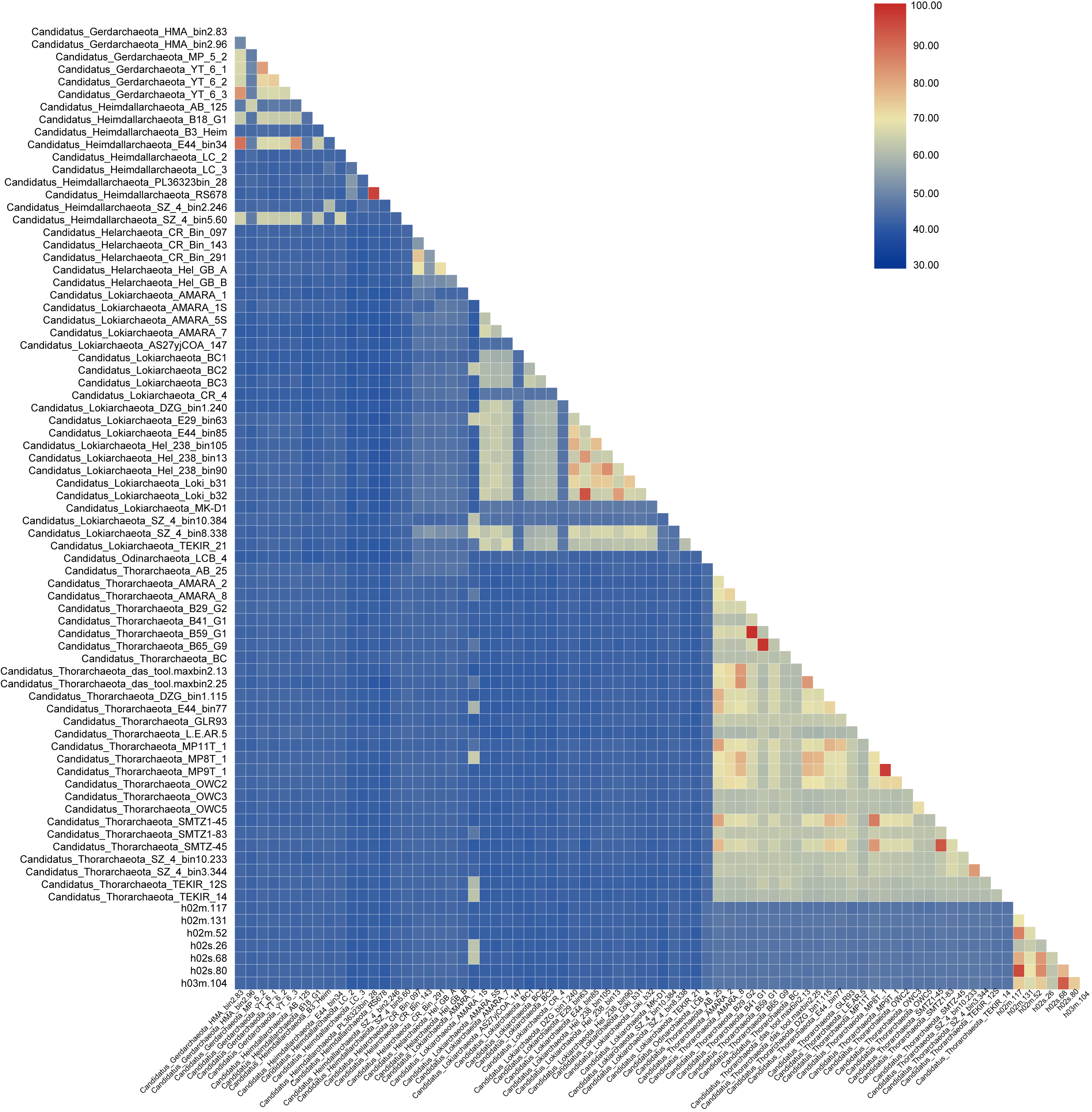
**

**Supplementary Fig. 4** Comparison of average amino acid identity (AAI) between Hermodarchaeota bins and published Asgard genomes. AAI was analyzed by CompareM.

**
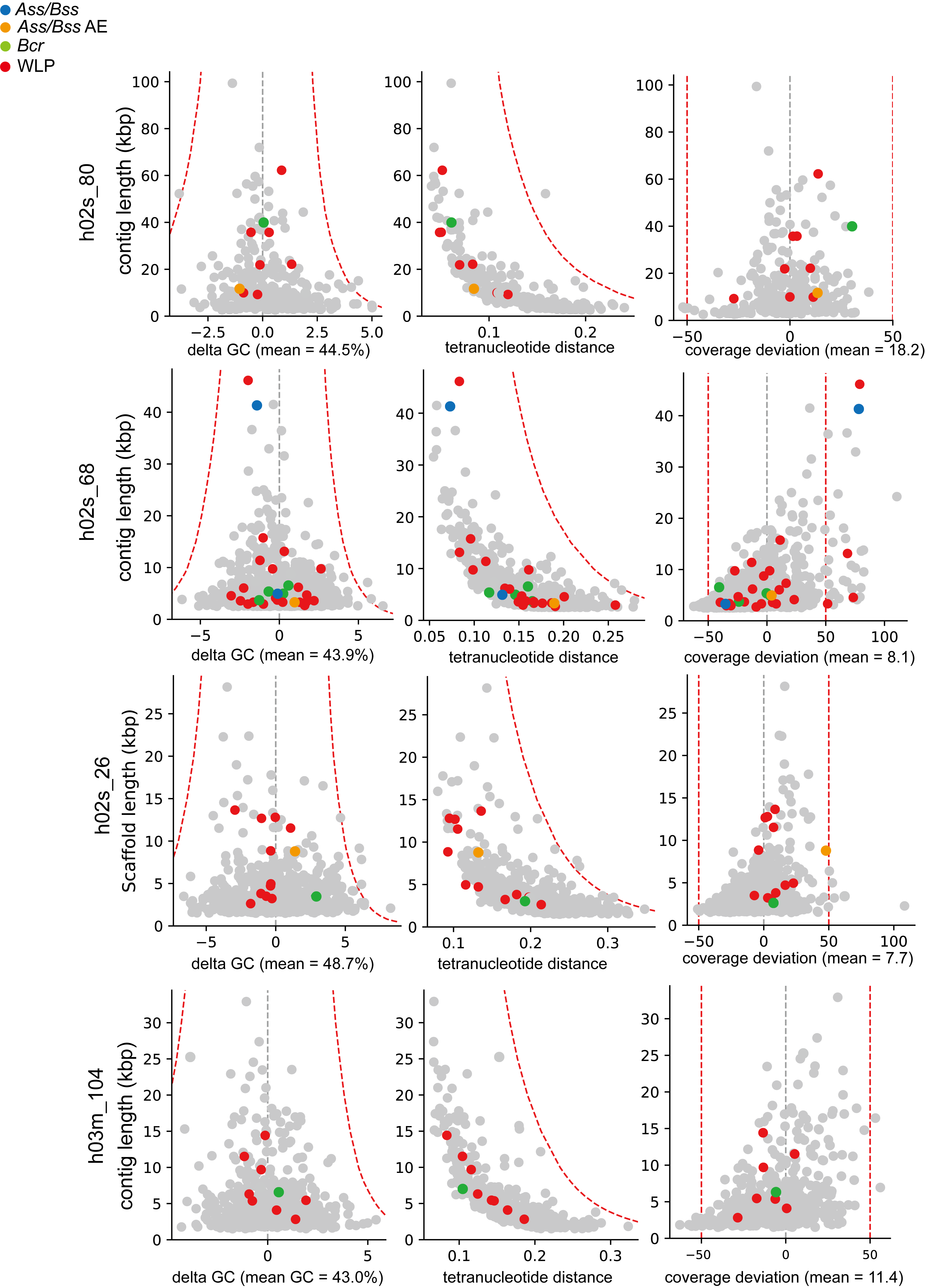
**

**Supplementary Fig. 5** Statistical characteristics of contigs constituting h02s_80, h02s_68, h02s_26 and h03m_104 genomes. The contig length was plotted as a function of the GC deviation, tetranucleotide distance and coverage deviation. Each dot in these scatterplots represents a contig. contigs containing *Ass/Bss* gene were labeled with blue; contigs containing *Ass/Bss* AE gene were labeled with orange; contigs containing *bcr* genes were labeled with green; red dots are contigs with genes for WLP. The dashed red lines indicate the 95^th^ percentile of a typical genome computed with RefineM. *Ass/Bss,* alkyl/benzylsuccinate synthase; *Ass/Bss* AE, Ass/ Bss-activating enzyme; Bcr, benzoyl-CoA reductase; WLP, Wood–Ljungdahl pathway.


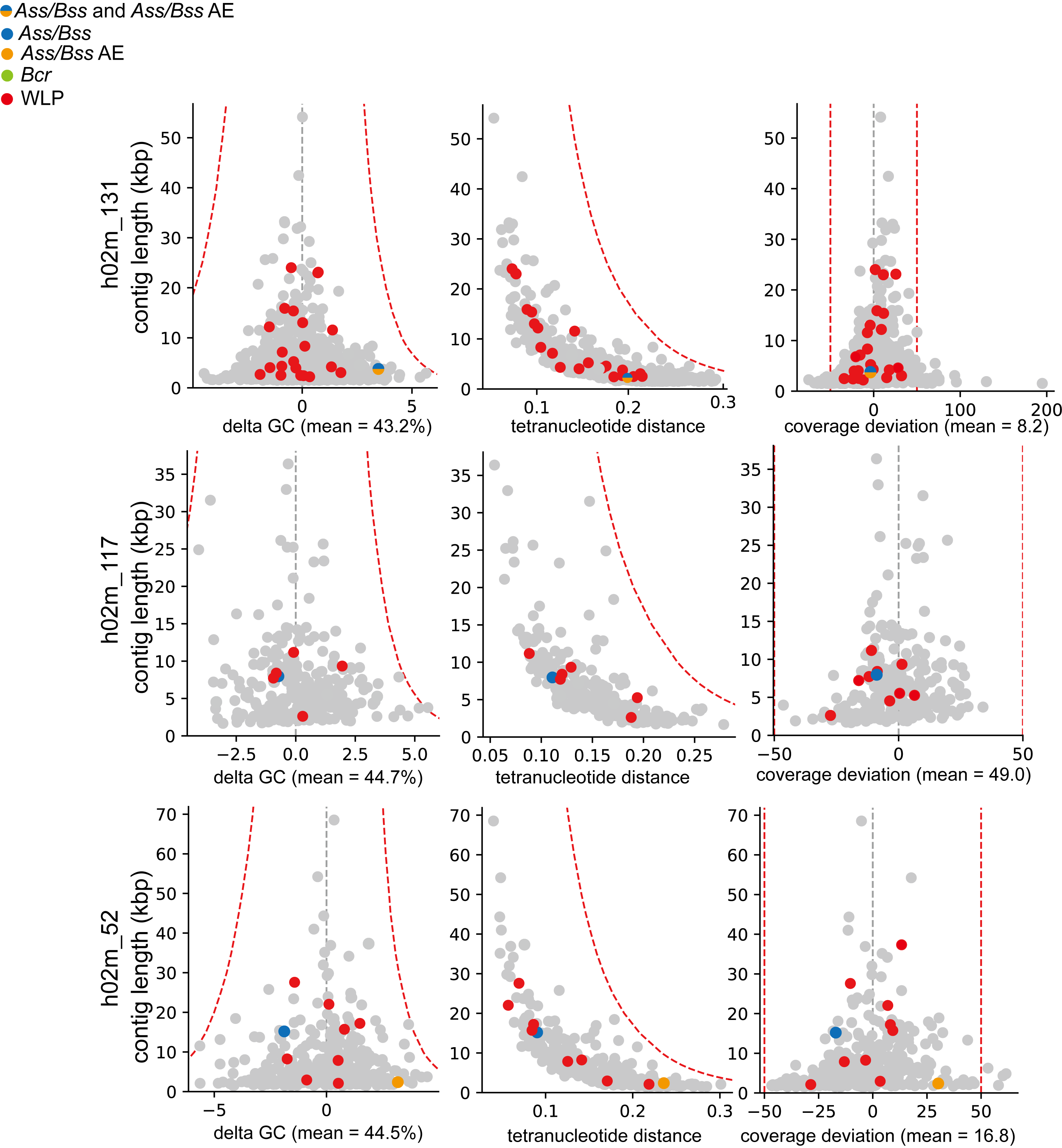


**Supplementary Fig. 6** Statistical characteristics of contigs constituting h02m_131, h02m_117, and h02m_52 genomes. The contig length was plotted as a function of the GC deviation, tetranucleotide distance and coverage deviation. Each dot in these scatterplots represents a contig. contigs containing *Ass/Bss* gene were labeled with blue; contigs containing *Ass/Bss* AE gene were labeled with orange; contigs containing *bcr* genes were labeled with green; red dots are contigs with genes for WLP; contigs containing both *Ass/Bss* and *Ass/Bss* AE genes were shaded in double colors. The dashed red lines indicate the 95^th^ percentile of a typical genome computed with RefineM. *Ass/Bss,* alkyl/benzylsuccinate synthase; *Ass/Bss* AE, Ass/Bss-activating enzyme; Bcr, benzoyl-CoA reductase; WLP, Wood–Ljungdahl pathway.


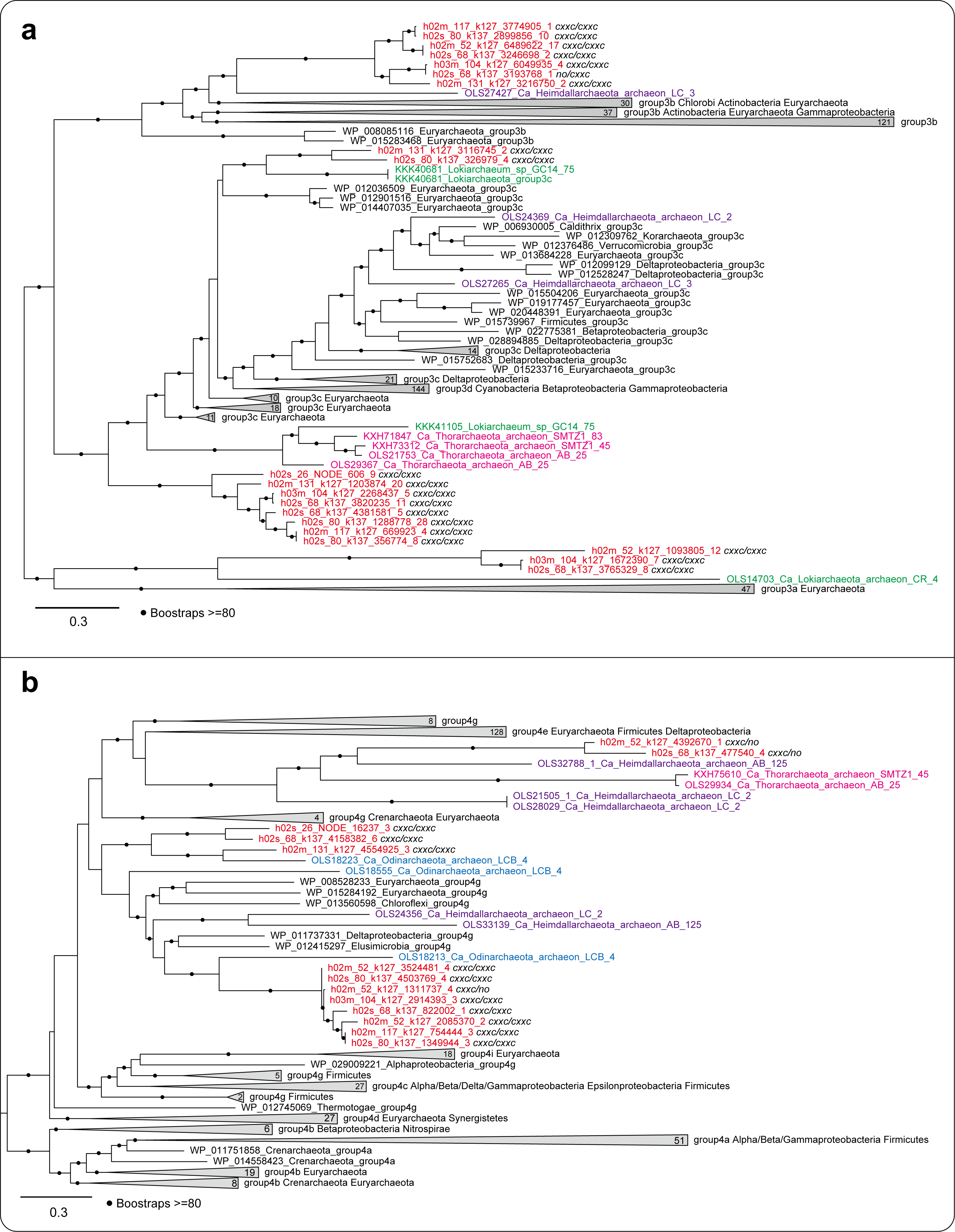


**Supplementary Fig. 7** Maximum-likelihood trees of the large subunit of group 3 and group 4 [NiFe]-hydrogenases reconstructed using IQtree v.1.6.12 with the best-fit model. **a** the large subunit of group 3 [NiFe]-hydrogenase (>300 amino acids); **b** the large subunit of group 4 [NiFe]-hydrogenase (>300 amino acids). Asgard hydrogenases were shaded in color as follows: Hermodarchaeota in red, Heimdallarchaeota in purple, Lokiarchaeota in green, Thorarchaetota in pink, and Odinarchaeota in blue. The N-terminal and C-terminal CxxC motifs were shown (two motifs: CxxC/ CxxC, one motif: no/ CxxC or CxxC/ no). Nodes with ultrafast bootstrap values ≥ 80 are indicated by black circles.

**
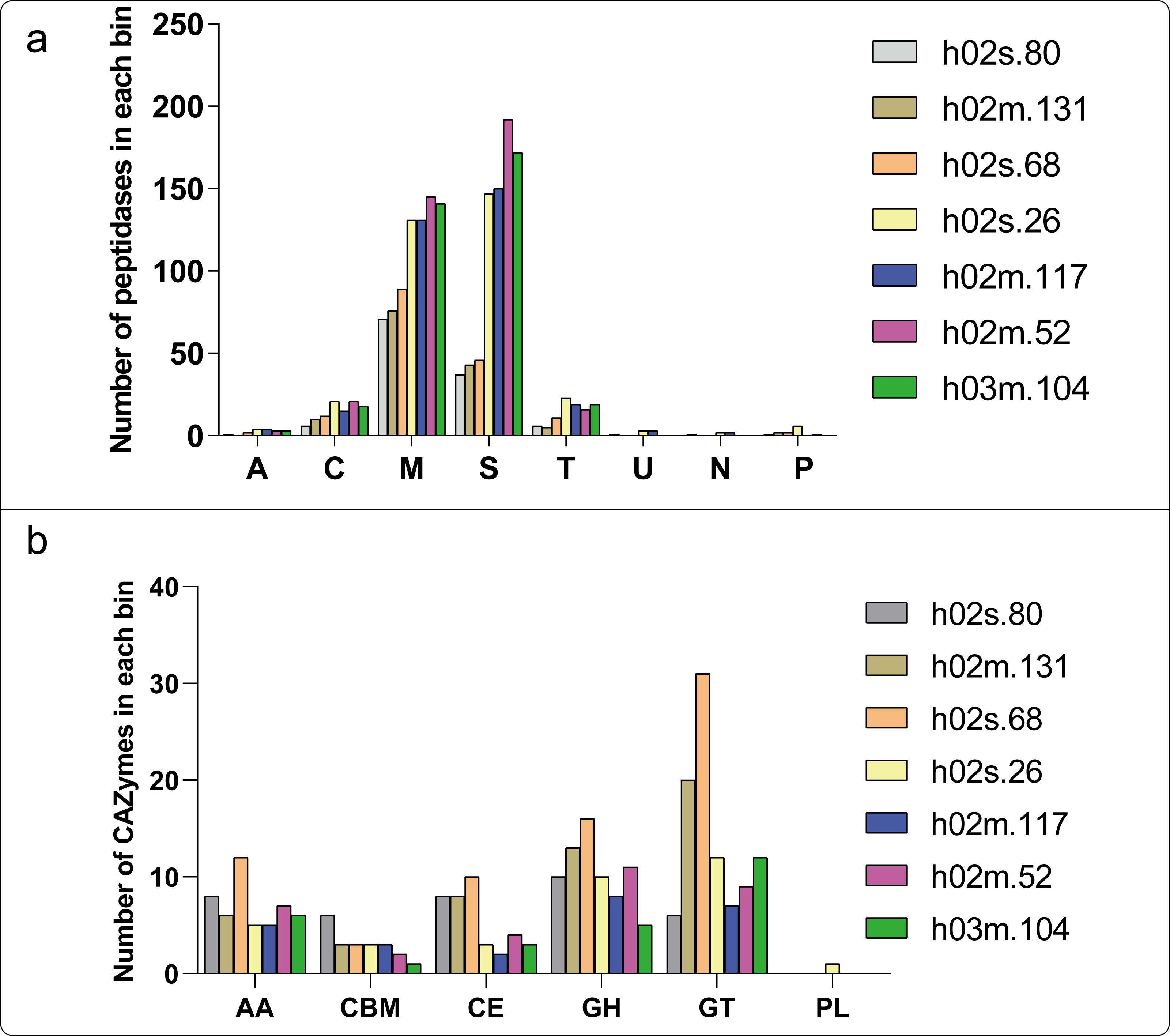
**

**Supplementary Fig. 8** Number of peptidases (a) and carbohydrate-active enzymes (CAZymes) (b) in Hermodarchaeota genomes. Peptidases and CAZymes were identified using MEROPS database and dbCAN web server using default parameters, respectively. A aspartic peptidase; C, cysteine peptidase; M, metallopeptidase; S, serine peptidase; T, threonine peptidase; U, unknown catalytic type; N, asparagine peptide lyase; P, mixed peptidase. AA, auxiliary activity; CBM, carbohydrate-binding module; CE, carbohydrate esterase; GH, glycoside hydrolase; GT, glycosyltransferase; PL, polysaccharide lyase.


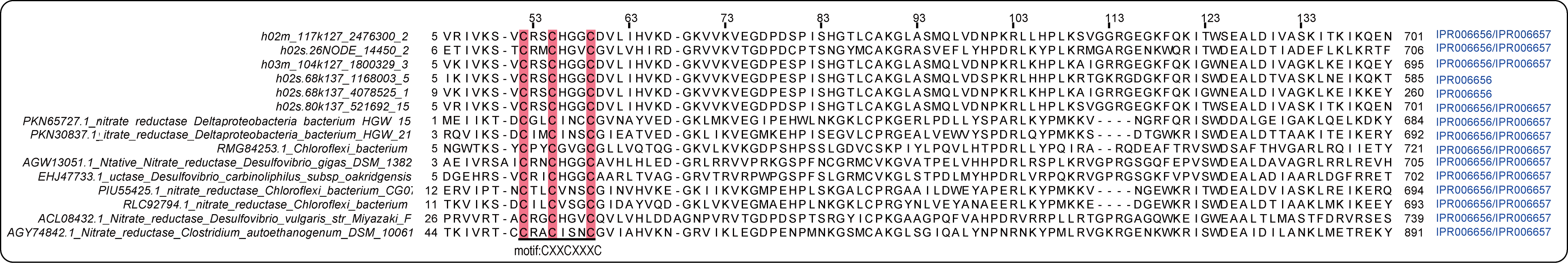


**Supplementary Fig. 9** Multiple sequence alignment of Hermodarchaeota nitrate reductases and homologues from nr database. These sequences contained a molybdopterin oxidoreductase domain (IPR006656) and molybdopterin guanine dinucleotide-binding (MGD) domain (IPR006657). The CXXCXXXC motif which is required for nitrate reductase is shown.

**
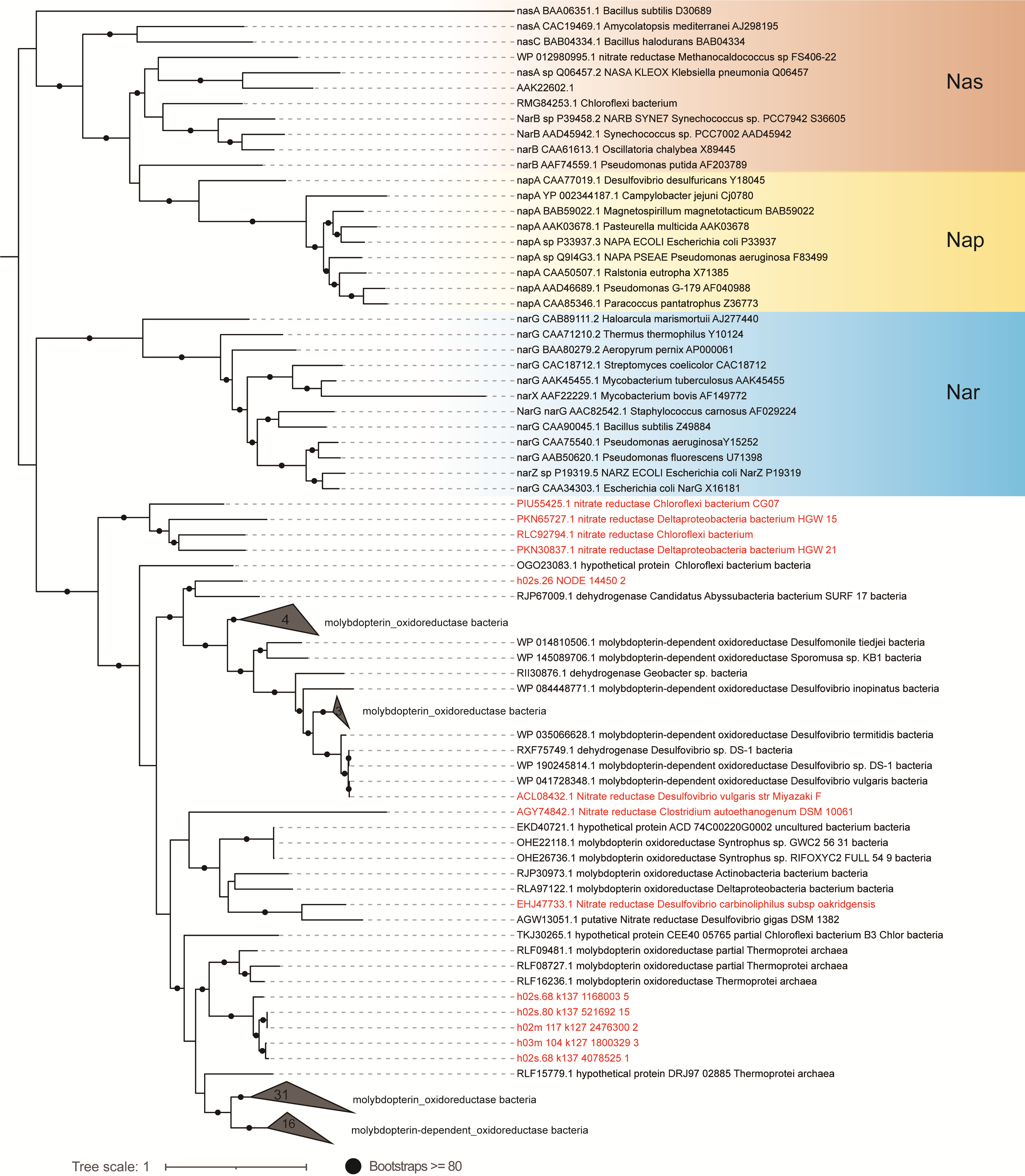
**

**Supplementary Fig. 10** Phylogenetic placement of nitrate reductases identified in Hermodarchaeota genomes. Maximum-likelihood tree was reconstructed using IQtree with under LG+ I+ G4 substitution model. A set of homologues of Nar, Nap and Nas from representative prokaryotic organisms were derived from a previous study [34]. The top 100 Hermodarchaeota nitrate reductase homologues in nr database were included in the tree. Putative nitrate reductases from Hermodarchaeota and other organisms were red-coded. Nas, prokaryotic assimilatory nitrate reductase; Nap, the periplasmic nitrate reductase; Nar, the membrane-associated prokaryotic nitrate reductase. Nodes with ultrafast bootstrap values ≥ 80 are indicated by black circles.

**
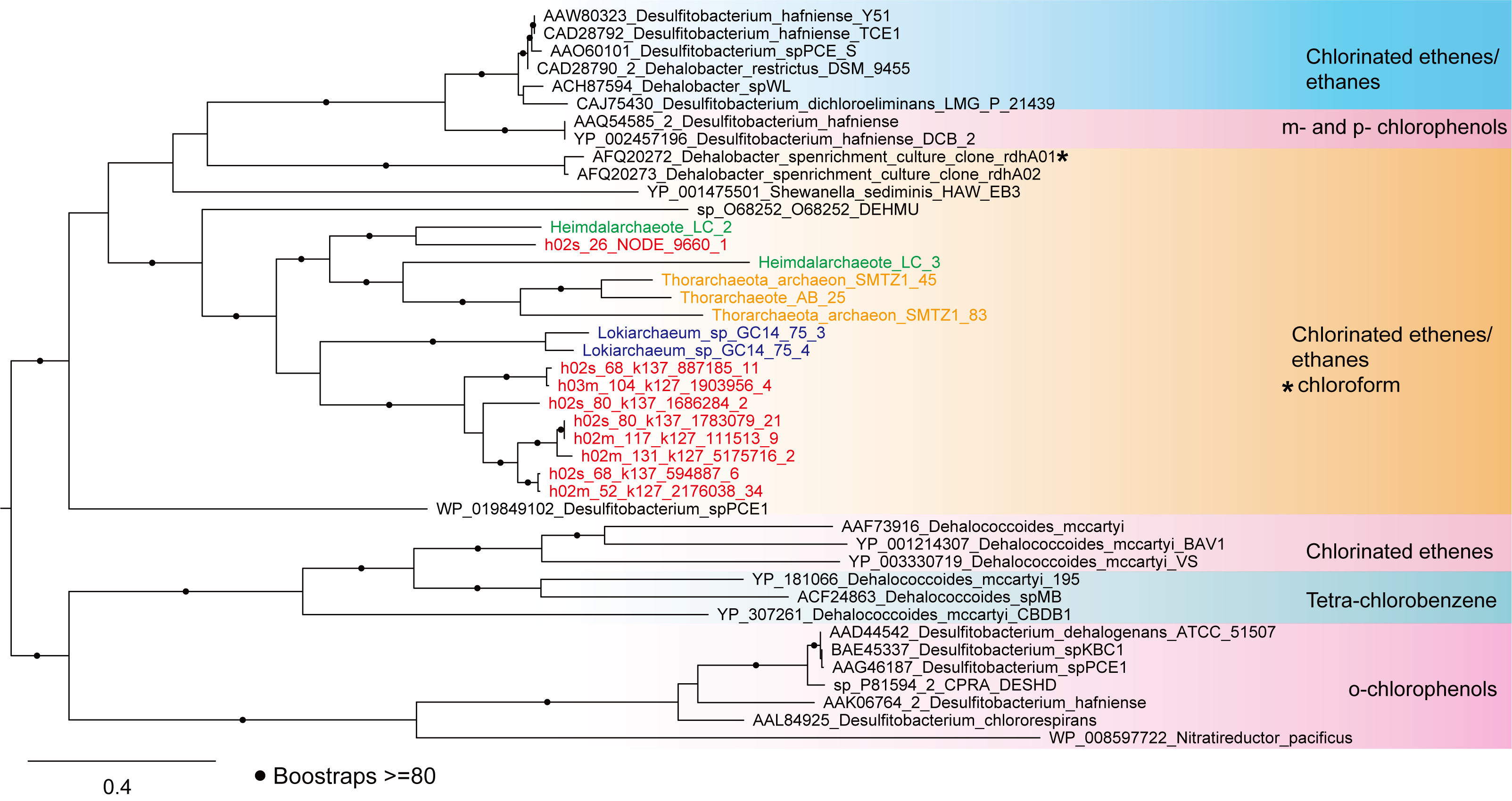
**

**Supplementary Fig. 11** Phylogenetic placement of reductive dehalogenases identified in Hermodarchaeota genomes. Functionally characterized reductive dehalogenases from bacteria were derived from a previous study [29]. Maximum-likelihood tree was reconstructed using IQtree with LG+I+G4 model. Asgard reductive dehalogenases were shaded in color as follows: Hermodarchaeota in red, Lokiarchaeota in blue, Thorarchaetota in yellow, Heimdallarchaeota in green. Nodes with ultrafast bootstrap values ≥ 80 are indicated by black circles.


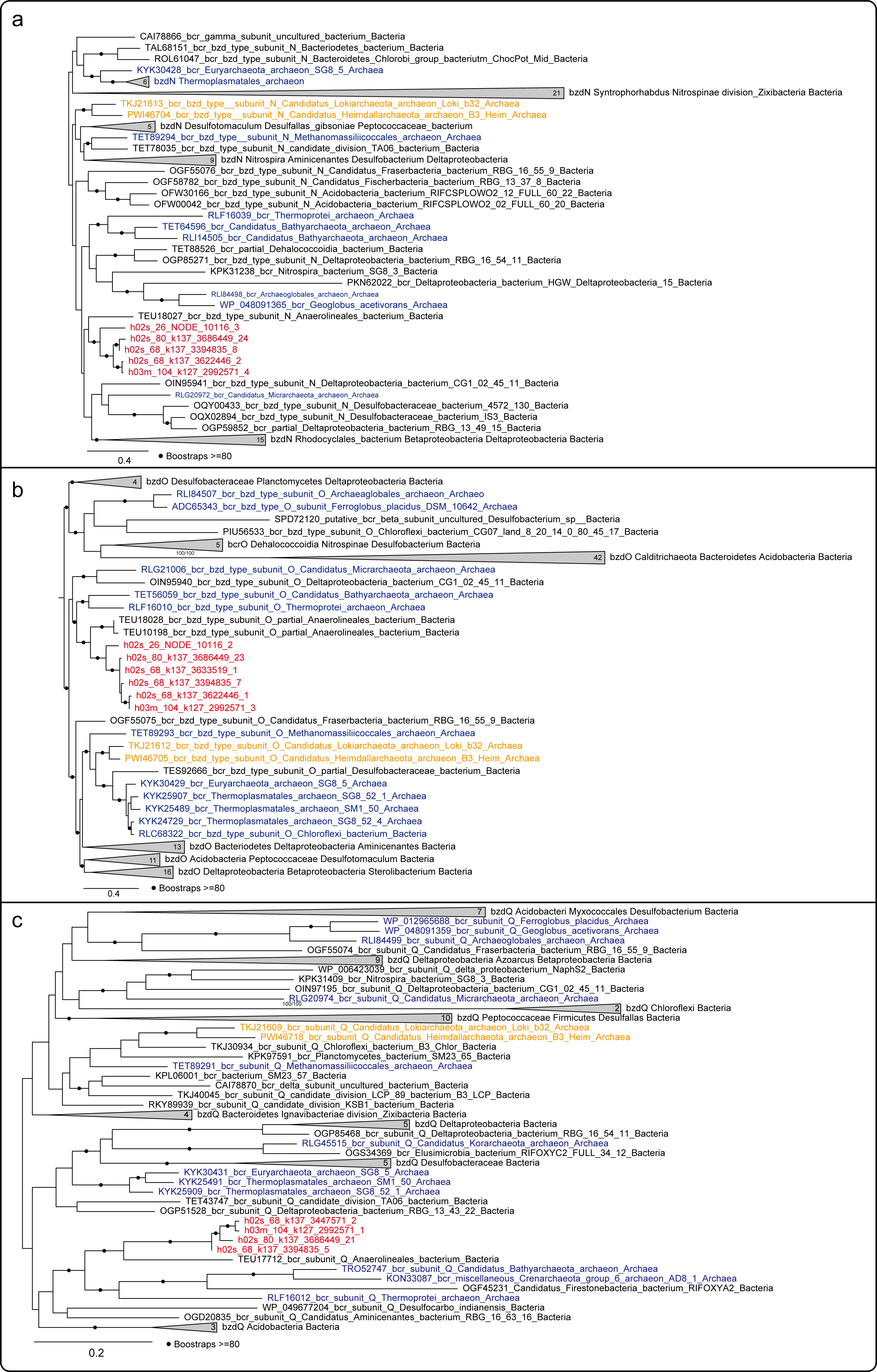


**Supplementary Fig. 12** Maximum-likelihood tree of benzoyl-CoA reductase (Bcr) identified in Hermodarchaeota genomes and homologues from nr database reconstructed using IQtree. Asgard archaeal benzoyl-CoA reductases were red-coded. **a** Benzoyl-CoA reductase subunit C (LG+G4 model); **b** Benzoyl-CoA reductase subunit B (LG+I+G4 model); **c** Benzoyl-CoA reductase subunit A (LG+I+G4 model). Nodes with ultrafast bootstrap values ≥ 80 are indicated by black circles.

**References**

1. Nguyen L-T, Schmidt HA, Von Haeseler A, Minh BQ. IQ-TREE: a fast and effective stochastic algorithm for estimating maximum-likelihood phylogenies. Mol Biol Evol. 2015;32:268-274.

2. Haft DH, Selengut JD, White O. The TIGRFAMs database of protein families. Nucleic Acids Res. 2003;31:371-373.

3. Finn RD, Bateman A, Clements J, Coggill P, Eberhardt RY, Eddy SR, et al. Pfam: the protein families database. Nucleic Acids Res. 2014;42:D222-D230.

4. Eddy SR. Accelerated profile HMM searches. PLoS Comput Biol. 2011;7.

5. Anantharaman K, Hausmann B, Jungbluth SP, Kantor RS, Lavy A, Warren LA, et al. Expanded diversity of microbial groups that shape the dissimilatory sulfur cycle. ISME J. 2018;12:1715-1728.

6. Tabita FR, Satagopan S, Hanson TE, Kreel NE, Scott SS. Distinct form I, II, III, and IV Rubisco proteins from the three kingdoms of life provide clues about Rubisco evolution and structure/function relationships. J Exp Bot. 2008;59:1515-1524.

7. Katoh K, Standley DM. MAFFT multiple sequence alignment software version 7: improvements in performance and usability. Mol Biol Evol. 2013;30:772-780.

8. Criscuolo A, Gribaldo S. BMGE (Block Mapping and Gathering with Entropy): a new software for selection of phylogenetic informative regions from multiple sequence alignments. BMC Evol Biol. 2010;10:210.

9. Li D, Liu C-M, Luo R, Sadakane K, Lam T-W. MEGAHIT: an ultra-fast single-node solution for large and complex metagenomics assembly via succinct de Bruijn graph. Bioinformatics. 2015;31:1674-1676.

10. Hyatt D, Chen G-L, LoCascio PF, Land ML, Larimer FW, Hauser LJ. Prodigal: prokaryotic gene recognition and translation initiation site identification. BMC Bioinf. 2010;11:119.

11. Altschul SF, Madden TL, Schäffer AA, Zhang J, Zhang Z, Miller W, et al. Gapped BLAST and PSI-BLAST: a new generation of protein database search programs. Nucleic Acids Res. 1997;25:3389-3402.

12. Fu L, Niu B, Zhu Z, Wu S, Li W. CD-HIT: accelerated for clustering the next-generation sequencing data. Bioinformatics. 2012;28:3150-3152.

13. Khelifi N, Ali OA, Roche P, Grossi V, Brochier-Armanet C, Valette O, et al. Anaerobic oxidation of long-chain n-alkanes by the hyperthermophilic sulfate-reducing archaeon, *Archaeoglobus fulgidus*. ISME J. 2014;8:2153-2166.

14. Merino F, Guixé V. Specificity evolution of the ADP‐dependent sugar kinase family–in silico studies of the glucokinase/phosphofructokinase bifunctional enzyme from Methanocaldococcus jannaschii. FEBS J. 2008;275:4033-4044.

15. Spang A, Stairs CW, Dombrowski N, Eme L, Lombard J, Caceres EF, et al. Proposal of the reverse flow model for the origin of the eukaryotic cell based on comparative analyses of Asgard archaeal metabolism. Nat Microbiol. 2019;4:1138-1148.

16. Say RF, Fuchs G. Fructose 1, 6-bisphosphate aldolase/phosphatase may be an ancestral gluconeogenic enzyme. Nature. 2010;464:1077-1081.

17. Adam PS, Borrel G, Gribaldo S. Evolutionary history of carbon monoxide dehydrogenase/acetyl-CoA synthase, one of the oldest enzymatic complexes. Proc Natl Acad Sci USA. 2018;115:E1166-E1173.

18. Berg IA, Kockelkorn D, Ramos-Vera WH, Say RF, Zarzycki J, Hügler M, et al. Autotrophic carbon fixation in archaea. Nat Rev Microbiol. 2010;8:447-460.

19. Borrel G, Adam PS, Gribaldo S. Methanogenesis and the Wood–Ljungdahl pathway: an ancient, versatile, and fragile association. Genome Biol Evol. 2016;8:1706-1711.

20. Harms U, Weiss DS, Gärtner P, Linder D, Thauer RK. The energy conserving N5‐methyltetrahydromethanopterin: coenzyme M methyltransferase complex from Methanobacterium thermoautotrophicum is composed of eight different subunits. Eur J Biochem. 1995;228:640-648.

21. Marreiros BC, Batista AP, Duarte AM, Pereira MM. A missing link between complex I and group 4 membrane-bound [NiFe] hydrogenases. Biochim Biophys Acta, Bioenerg. 2013;1827:198-209.

22. Bäumer S, Ide T, Jacobi C, Johann A, Gottschalk G, Deppenmeier U. The F420H2 Dehydrogenase fromMethanosarcina mazei Is a Redox-driven Proton Pump Closely Related to NADH Dehydrogenases. J Biol Chem. 2000;275:17968-17973.

23. Hedderich R. Energy-converting [NiFe] hydrogenases from archaea and extremophiles: ancestors of complex I. J Bioenerg Biomembr. 2004;36:65-75.

24. Peters JW, Schut GJ, Boyd ES, Mulder DW, Shepard EM, Broderick JB, et al. [FeFe]-and [NiFe]-hydrogenase diversity, mechanism, and maturation. Biochim Biophys Acta, Mol Cell Res. 2015;1853:1350-1369.

25. Schut GJ, Zadvornyy O, Wu C-H, Peters JW, Boyd ES, Adams MW. The role of geochemistry and energetics in the evolution of modern respiratory complexes from a proton-reducing ancestor. Biochim Biophys Acta, Bioenerg. 2016;1857:958-970.

26. Yu H, Wu C-H, Schut GJ, Haja DK, Zhao G, Peters JW, et al. Structure of an ancient respiratory system. Cell. 2018;173:1636-1649. e1616.

27. Adrian L, Szewzyk U, Wecke J, Görisch H. Bacterial dehalorespiration with chlorinated benzenes. Nature. 2000;408:580-583.

28. Bunge M, Adrian L, Kraus A, Opel M, Lorenz WG, Andreesen JR, et al. Reductive dehalogenation of chlorinated dioxins by an anaerobic bacterium. Nature. 2003;421:357-360.

29. Payne KA, Quezada CP, Fisher K, Dunstan MS, Collins FA, Sjuts H, et al. Reductive dehalogenase structure suggests a mechanism for B12-dependent dehalogenation. Nature. 2015;517:513-516.

30. Kristensen E, Bouillon S, Dittmar T, Marchand C. Organic carbon dynamics in mangrove ecosystems: a review. Aquat Bot. 2008;89:201-219.

31. Zhichao, Zhou, Yang, Liu, Karen, Lloyd, et al. Genomic and transcriptomic insights into the ecology and metabolism of benthic archaeal cosmopolitan, Thermoprofundales (MBG-D archaea). ISME J. 2019.

32. Deppenmeier U, Johann A, Hartsch T, Merkl R, Schmitz RA, Martinez-Arias R, et al. The genome of Methanosarcina mazei: evidence for lateral gene transfer between bacteria and archaea. J Mol Microbiol Biotechnol. 2002;4:453-461.

33. Fricke WF, Seedorf H, Henne A, Krüer M, Liesegang H, Hedderich R, et al. The genome sequence of Methanosphaera stadtmanae reveals why this human intestinal archaeon is restricted to methanol and H2 for methane formation and ATP synthesis. J Bacteriol. 2006;188:642-658.

34. Stolz JF, Basu P. Evolution of nitrate reductase: molecular and structural variations on a common function. Chembiochem. 2002;3:198-206.
